# Emotional intelligence and holistic student development: an assessment of psychological and social efficacy in vocational university English education

**DOI:** 10.3389/fpsyg.2025.1664645

**Published:** 2025-12-18

**Authors:** Qilin Xuan

**Affiliations:** Jiujiang Polytechnic University of Science and Technology, Jiujiang, Jiangxi, China

**Keywords:** emotional intelligence (EI), university English education, psychological well-being, social competence, learning development

## Abstract

The role of Emotional Intelligence (EI) in university English education has garnered increasing attention, particularly regarding its impact on students’ psychological well-being and social competencies. This study explores how EI influences university students’ psychological adaptability, interpersonal communication skills, and overall learning experience. Employing a mixed-methods approach, this research integrates quantitative surveys and qualitative in-depth interviews to assess the EI levels and their psychological and social efficacy among university English learners at Jiujiang Polytechnic University of Science and Technology. Data analysis reveals a significant correlation between higher EI levels and better mental health, enhanced social interaction skills, and a more positive learning attitude. Through the integration of EI training within university English courses, students exhibit improved self-regulation and teamwork abilities. Furthermore, this study underscores the necessity of incorporating EI into university English teaching and provides both theoretical foundations and practical recommendations for future curriculum design and pedagogical strategies. The findings offer valuable insights for English educators, mental health professionals, and educational policymakers.

## 1 Introduction

### 1.1 Research background and significance

In the context of rapid globalization and digital transformation, university English education transcends mere linguistic skill development, serving as a critical avenue for fostering students’ psychological well-being, social adaptability, and holistic growth (Rajaram, 2023; Özyurt and Kalgı, 2024; Kayyali, 2024). In recent years, Emotional Intelligence (EI) has emerged as a key determinant of personal development and social competence, drawing increasing attention from educational researchers (Muñoz-Oliver et al., 2022; Rico-Gonzalez, 2023; Antonopoulou, 2024; Gazi et al., 2024). Goleman (1995) posited that EI comprises multiple dimensions, including self-awareness, self-regulation, social skills, and empathy, all of which not only influence academic performance but also exert profound effects on students’ mental health and interpersonal relationships.

Within university English classrooms, students frequently encounter challenges such as Foreign Language Anxiety (FLA), lack of confidence, and social barriers (Akaraphattanawong et al., 2024; Özdemir and Seçkin, 2025). Cultivating EI has been shown to mitigate these issues by enhancing learners’ emotional regulation and social adaptation abilities. Researches indicate that students with higher EI demonstrate greater resilience and motivation in foreign language learning and are more adept at leveraging social support systems to facilitate their studies (Namaziandost and Rezai, 2024; Tingyu et al., 2024). Given these considerations, investigating the role of EI in university English education—particularly its effects on students’ psychological well-being and social competencies—holds significant theoretical and practical value (Shengyao et al., 2024; Zong and Yang, 2025). This study aims to assess the relationship between students’ EI levels, mental health, and social abilities while exploring effective strategies for integrating EI training into university English classrooms to foster holistic student development.

### 1.2 The significance of emotional intelligence in education

In recent years, the growing significance of EI in education has become increasingly evident (Khassawneh et al., 2022; Wiedermann et al., 2023; Hashmi and Bal, 2024). Educational psychology research has highlighted that traditional academic assessments often overemphasize cognitive abilities while overlooking the essential role of non-cognitive factors in student development (Sultanova and Shora, 2024). Salovey and Mayer (1990) initially introduced the concept of EI, defining its core competencies as the ability to perceive, understand, and regulate emotions—skills that significantly impact learners’ motivation, academic performance, teamwork, and psychological well-being. In university English education, students’ EI not only influences their use of language learning strategies but is also closely linked to their cross-cultural communication competence (Gao et al., 2025).

The positive impact of EI on psychological well-being has been extensively documented (Ansari et al., 2024). Individuals with high EI are better equipped to regulate anxiety, respond proactively to academic stress, and thereby reduce the prevalence of mental health issues such as depression and anxiety (Augusto-Landa et al., 2024; Sun M, et al., 2024). Moreover, EI profoundly influences students’ social competencies (Ida Merlin and Prabakar, 2024; Imjai et al., 2024). Learners with strong EI are more likely to establish positive interpersonal relationships, exhibit greater social adaptability in classroom collaboration and language exchanges, and, consequently, enhance their learning outcomes (Krishna et al., 2024; Yan et al., 2024).

Within university English teaching contexts, fostering students’ EI contributes to improved classroom interaction, strengthened autonomous learning capabilities, and the promotion of positive learning attitudes (Sadiqzade, 2024; Namaziandost et al., 2024). Therefore, integrating EI into university English curricula aligns with contemporary educational development trends while offering a novel perspective for cultivating well-rounded individuals with robust psychological resilience and social adaptability (Ayeni et al., 2024). This study further examines the application of EI theories in university English classrooms to improve students’ mental well-being and social competence, ultimately fostering comprehensive personal growth.

## 2 Literature review

### 2.1 Theoretical foundations of emotional intelligence

#### 2.1.1 Definition and development of emotional intelligence

Emotional Intelligence (EI) has been widely conceptualized as the capacity to perceive, understand, regulate, and utilize emotions in ways that facilitate adaptive functioning in both personal and social domains (Salovey and Mayer, 1990; Mayer et al., 2004). While traditional conceptualizations of intelligence emphasized cognitive ability as the primary determinant of academic achievement, subsequent research has demonstrated that affective and interpersonal competencies exert equally decisive influence, particularly in educational contexts (Pekrun, 2006). Building on this trajectory, Goleman (1995) expanded the conceptual scope of EI by emphasizing its broader applicability to social competence, workplace performance, and professional development, thereby framing EI as a set of skills that enable individuals to navigate emotional experiences, sustain interpersonal relationships, and achieve optimal outcomes.

In recent years, with the growth of educational psychology and cognitive sciences, the role of EI in learning and teaching has garnered substantial scholarly attention (Gkintoni et al., 2021; Wang et al., 2022; Drigas et al., 2023; Liu and Chang, 2024). Empirical evidence consistently indicates that EI not only predicts academic performance but also contributes to psychological well-being, resilience, and social adaptability (MacCann et al., 2020; Dewaele et al., 2019; Guillen K. P. et al., 2022; Guillen M. E. et al., 2022; Gkintoni et al., 2023; Sadiqzade, 2024). Cross-cultural investigations further suggest that university students with higher levels of EI demonstrate superior coping strategies in response to academic stress and display stronger adaptability in challenging environments (González-Martín et al., 2023). Similarly, research in higher education has established close links between EI and classroom engagement, social interaction skills, and long-term academic persistence (Zhoc et al., 2023; Han et al., 2022).

Within the domain of second and foreign language learning, EI has emerged as a critical variable influencing learners’ affective experiences and communicative competence. Studies have shown that EI correlates strongly with language-related constructs such as foreign language anxiety (FLA), motivation, and self-efficacy (Dewaele and MacIntyre, 2022). This growing body of literature has inspired scholars to explore pedagogical approaches for cultivating EI in classroom settings, thereby helping learners better navigate the affective and interpersonal challenges of language acquisition (Thao et al., 2023; Li and Zhang, 2024).

Nevertheless, the majority of existing studies remain situated in general university contexts, with relatively limited attention to vocational education. Vocational colleges, in contrast, emphasize practice-oriented, skill-based training that requires students to demonstrate adaptability, communication competence, and resilience in workplace-simulated environments. In this regard, the present study advances the theoretical application of EI by positioning it as a pivotal construct that bridges academic learning with professional competence. This reconceptualization moves beyond a purely academic orientation toward a practice-oriented perspective, thereby offering a novel theoretical contribution to the integration of EI into vocational English education.

#### 2.1.2 Comparison of Goleman’s and Mayer-Salovey’s models

Research on Emotional Intelligence (EI) has been primarily shaped by two dominant frameworks: the ability model developed by Mayer and Salovey (1997) and the mixed model advanced by Goleman (1995, 1998). The Mayer-Salovey model conceptualizes EI as a set of cognitive-emotional abilities, including the perception, understanding, regulation, and management of emotions, and views EI as a measurable psychological construct independent of traditional intelligence (Mayer et al., 2016). In contrast, Goleman’s model emphasizes the applied dimensions of EI in social and professional contexts, categorizing it into five interrelated domains: self-awareness, self-regulation, motivation, empathy, and social skills (Boyatzis et al., 2023).

While the Mayer-Salovey model provides a rigorous cognitive foundation by explaining how individuals process and regulate emotional information, Goleman’s framework broadens the scope by incorporating behavioral competencies essential for interpersonal effectiveness and professional success. Recent studies have underscored the complementarity of these models, with empirical evidence indicating that Mayer-Salovey-based interventions are particularly effective in enhancing cognitive regulation, whereas Goleman’s framework excels in fostering teamwork, communication, and leadership abilities (Coronado-Maldonado and Benítez-Márquez, 2023; Bhaal et al., 2024; Sharma and Pandey, 2024; Sakyi and Korang, 2024).

In the context of vocational English education, these two models are best understood as synergistic rather than mutually exclusive. The Mayer-Salovey model provides insights into the cognitive-emotional mechanisms underlying language learning, such as reducing foreign language anxiety and sustaining classroom engagement. At the same time, Goleman’s framework highlights the interpersonal and professional dimensions—teamwork, adaptability, and communication—that align closely with the practice-oriented goals of vocational education. By integrating both perspectives, this study develops a hybridized conceptual framework that captures EI’s dual role: supporting linguistic performance while simultaneously cultivating transferable soft skills for workplace readiness.

### 2.2 Relationship between language learning and mental health

#### 2.2.1 Foreign language anxiety

Foreign Language Anxiety (FLA) is widely acknowledged as a central construct in understanding the psychological barriers to second language acquisition. Initially defined by Horwitz et al. (1986), FLA encompasses communication apprehension, test anxiety, and fear of negative evaluation. While these three dimensions provide a useful typology, they have sometimes been criticized for overlapping in practice, as learners’ apprehension in communication is often intertwined with evaluative concerns in both peer and teacher interactions. This overlap suggests that the construct may be more dynamic and context-dependent than the original framework implies.

Empirical evidence has reinforced the negative impact of FLA on language learning. For instance, Dewaele and MacIntyre (2022) demonstrated that heightened levels of FLA significantly undermine oral expression and listening comprehension, ultimately eroding students’ classroom confidence. However, this perspective risks overemphasizing anxiety as a deficit, neglecting the possibility that moderate anxiety may, in certain contexts, motivate greater preparation or effort. More recent studies provide a more nuanced understanding by showing that EI training can alleviate FLA, which points toward a proactive, skill-based intervention model rather than treating anxiety as an immutable trait (Han et al., 2022; Liu and Wang, 2023).

In addition, Fu (2024) findings underscore that learners with higher EI are not merely less anxious but also more resilient, suggesting that the interaction between EI and FLA should be conceptualized as reciprocal: while EI reduces anxiety, experiences of managing anxiety in turn foster EI-related growth.

#### 2.2.2 Self-efficacy in language learning

Self-efficacy, introduced by Bandura (1986), refers to individuals’ judgments about their ability to perform specific tasks successfully. In language learning, self-efficacy not only shapes students’ motivational orientations but also influences the strategies they adopt and their persistence when encountering difficulties (Graham, 2022). High levels of self-efficacy are consistently linked to stronger autonomous learning capacity, active participation, and communicative confidence (Ismail et al., 2023). Nonetheless, the literature tends to treat self-efficacy as a stable trait, underexploring the situational fluctuations that occur in multilingual classrooms, where learners’ confidence often varies across tasks (e.g., speaking vs. writing) and over time.

EI has been increasingly recognized as a critical factor in enhancing self-efficacy. Han et al. (2022) confirmed a positive association between EI and self-efficacy, indicating that emotion regulation skills allow learners to transform anxiety into productive engagement. Importantly, Al-khresheh and Alkursheh’s (2024) empirical research extends this line of inquiry by showing that EI training not only boosts language performance across multiple modalities but does so precisely by strengthening learners’ confidence in their capacity to manage communicative challenges. This finding underscores that self-efficacy is not merely an outcome but also a mediating mechanism through which EI exerts its influence on language achievement.

From a pedagogical perspective, interventions such as constructive feedback, the creation of psychologically safe classroom environments, and structured emotional management training have been recommended (Gilar-Corbi et al., 2024). Yet, these strategies may need to be further tailored for vocational education contexts, where the emphasis on practical communication skills and workplace readiness imposes different demands compared to general academic learning. Thus, future studies should examine whether the mechanisms linking EI and self-efficacy operate similarly in skill-oriented, professional English training, or whether new contextual variables must be incorporated.

### 2.3 The role of social competence in university-level English learning

#### 2.3.1 Impact of social competence on language acquisition

Social competence is a vital determinant of successful second language acquisition, as language learning is inherently embedded in interactional contexts. Vygotsky’s (1978) Sociocultural Theory conceptualizes language development as a socially mediated process, wherein learners construct meaning through dialogic engagement with peers and instructors (Alkhudiry, 2022). However, while this theoretical foundation emphasizes the universality of social interaction, more recent scholarship has highlighted the uneven ways in which social competence manifests in classrooms, depending on learners’ cultural backgrounds, prior schooling, and personality traits. This suggests that social competence, though broadly facilitative, is not uniformly distributed and may interact with contextual variables such as classroom culture and institutional environment.

Empirical findings underscore its significance. Recent studies indicate that socially competent students are more willing to participate in classroom exchanges, form interpersonal bonds, and use the target language beyond formal tasks (Dewaele and Li, 2021; Dai, 2023; Hu and Wang, 2023; Rashov, 2024). Bordeos et al. (2023) further confirm that these students often demonstrate superior oral fluency and grammatical accuracy, not merely because of inherent ability, but due to the expanded communicative opportunities that active participation affords. Yet, these findings raise a critical question: to what extent does social competence function as a cause of improved proficiency versus an outcome of greater linguistic confidence? Nkhi (2022) observation that socially adept learners maintain composure in uncertain communicative situations suggests a reciprocal relationship, where competence both facilitates and is reinforced by communicative success.

The role of EI in this process is increasingly acknowledged. Abbas (2023) demonstrated that EI training enhances empathy and self-regulation, two skills central to building social competence and navigating group dynamics. This finding suggests that social competence should not be viewed solely as a precondition for language learning but as a malleable capacity that can be intentionally fostered through emotional skill-building. For vocational education contexts, where communicative competence often needs to be applied in professional and intercultural settings, such an EI-informed approach offers practical value that extends beyond academic classrooms.

#### 2.3.2 Classroom interaction and learning outcomes

Classroom interaction constitutes the primary site where language input is transformed into active competence. Lantolf (2000) Dynamic Assessment Theory highlights that learners progress within their Zone of Proximal Development (ZPD) when supported by feedback and collaborative scaffolding. Nevertheless, existing research often assumes that increased interaction automatically yields better outcomes, overlooking the possibility that low-quality or perfunctory interactions may reinforce surface-level learning rather than deep engagement. Thus, a more critical consideration lies in differentiating between “quantity” and “quality” of classroom interactions in shaping learning outcomes.

Jiang et al. (2022) argue that rich, dialogic classroom interactions improve comprehension and expressive skills, a claim further supported by studies linking EI to students’ interactive behavior. Sadiqzade (2024) observed that learners with higher EI actively participate in collaborative tasks, while Derakhshan et al. (2022) found that such interaction builds resilience against setbacks, suggesting that EI functions as a moderator of the interaction–achievement relationship. Yet, one limitation across these studies is the tendency to treat students as homogeneous actors, without fully examining whether high-EI learners dominate discussions and inadvertently marginalize others with lower EI, thereby reproducing inequities within the classroom.

Teachers’ EI also plays a critical role. Sun Y, et al. (2024) revealed that emotionally intelligent instructors are better at cultivating positive climates, reducing affective barriers, and encouraging broader participation. While this finding is promising, it raises a further tension: should the onus of social competence development lie primarily with students, or should it be seen as a shared responsibility between instructors and institutional design? Recognizing this interplay highlights a broader pedagogical imperative—that EI-informed teaching should focus not only on enhancing individual learners’ competencies but also on structuring interactional norms that ensure inclusivity and balanced participation.

Taken together, the literature suggests that social competence and EI mutually reinforce one another, shaping the quality of classroom interactions and learning outcomes. However, the challenge for university-level English teaching, particularly in vocational contexts, lies in designing interventions that translate this synergy into practical communicative skills aligned with workplace demands.

### 2.4 Conceptual framework

#### 2.4.1 Core dimensions of emotional intelligence

Emotional Intelligence (EI) is conceptualized as an individual’s capacity to perceive, comprehend, and regulate emotions within social interactions and learning contexts (Salovey and Mayer, 1990). Building upon this theoretical foundation, Goleman (1995) delineated EI into four fundamental dimensions, each of which exerts a distinctive influence on language learning.

To begin with, self-awareness refers to an individual’s ability to recognize and interpret their emotional states and understand the impact of these emotions on their behaviors and decision-making processes (Goleman, 1998). Within language acquisition, this dimension is critical because learners who can recognize anxiety or confidence more accurately are able to adjust their learning strategies. For example, a student aware of their speaking anxiety may adopt gradual exposure techniques to reduce stress. Without such awareness, negative emotions often go unchecked, leading to avoidance behaviors.

Self-regulation, in turn, enables students to adaptively manage their emotional responses (Mayer and Salovey, 1997). Its importance lies in transforming potentially detrimental emotions into productive ones. In practice, learners with strong self-regulation may channel exam-related stress into focused preparation, whereas those lacking this skill might experience cognitive overload. Thus, self-regulation acts as a protective factor that directly influences persistence and resilience in language classrooms.

Meanwhile, social skills are indispensable for building collaborative learning environments and sustaining interaction (Bar-On, 2006). Students with strong social skills not only participate more actively but also benefit from peer scaffolding, which enhances linguistic development. Conversely, limited social skills may isolate learners, reducing opportunities for authentic communication. This highlights why university-level English instruction must go beyond grammar and vocabulary to cultivate interpersonal abilities.

Finally, empathy fosters deeper interpersonal understanding (Goleman, 1995). In group-based learning and intercultural exchanges, empathy allows learners to appreciate different perspectives and adapt communicative styles accordingly. This dimension is especially relevant in multilingual classrooms, where the absence of empathy can reinforce cultural barriers instead of promoting mutual learning.

Moreover, motivation constitutes the fifth key dimension of Goleman’s mixed model, reflecting the internal drive to achieve goals beyond external rewards (Goleman, 1998; Goleman et al., 2013). In the context of language learning, motivated students demonstrate persistence in the face of difficulties, resilience after setbacks, and proactive engagement in communicative practice. Research shows that motivation is strongly associated with academic perseverance and achievement (Ryan and Deci, 2000; Dörnyei, 2005). Importantly, motivation does not function in isolation but interacts with other EI domains: self-awareness helps learners recognize their intrinsic goals, regulation sustains effort under stress, social skills foster collaborative encouragement, and empathy enriches culturally relevant learning. Therefore, motivation serves as a dynamic catalyst that integrates emotional capacities with purposeful academic action.

Taken together, these five EI dimensions are not isolated but mutually reinforcing. Self-awareness provides the foundation for regulation, regulation strengthens social skills, empathy enhances both interpersonal connection and collaborative learning, while motivation drives sustained effort and purposeful learning. Critically, this interdependence suggests that interventions targeting one EI domain may generate broader benefits for students’ emotional and academic development.

#### 2.4.2 Psychological well-being factors in language learning

Psychological well-being is a critical determinant of foreign language learning efficacy, with anxiety, self-confidence, and motivation being particularly influential (MacIntyre and Gregersen, 2012). However, these factors should not be viewed as static traits; instead, they operate dynamically and often interact in complex ways.

Language anxiety, for instance, is among the most pervasive challenges faced by learners (Horwitz et al., 1986). Excessive Foreign Language Anxiety (FLA) can paralyze learners’ ability to speak and process language input. Yet, it is worth noting that mild levels of anxiety can sometimes serve as a motivator, pushing students to prepare more thoroughly. This dual effect underscores the importance of regulation mechanisms, such as EI in distinguishing between facilitative and debilitative forms of anxiety.

Self-confidence functions as both a predictor and an outcome of successful language use (Bandura, 1997). Learners with higher confidence are more willing to take risks and engage in authentic communication. However, confidence without competence may lead to overestimation of ability, while low confidence undermines motivation despite adequate skills. Thus, confidence must be cultivated alongside feedback mechanisms that ensure its alignment with actual progress.

Motivation represents another central dimension (Dörnyei, 2005). Intrinsic motivation often predicts sustained effort, but motivation is highly susceptible to contextual influences, such as teacher support and classroom climate. For example, learners may initially be motivated by grades (extrinsic), but through positive classroom experiences, this can transform into intrinsic interest. Therefore, psychological well-being in language learning is best understood as a dynamic system shaped by personal, social, and instructional factors.

Crucially, EI underpins all three factors: it mitigates the negative effects of anxiety, stabilizes confidence, and sustains motivation. This integrative role suggests that cultivating EI is not an auxiliary goal but a central mechanism for fostering psychological well-being in language learning environments.

#### 2.4.3 Mechanisms by which social competence influences learning outcomes

Social competence is recognized as a crucial determinant of language learning success, exerting its influence primarily through classroom interactions and communicative exchanges (Swain, 2000). Yet, its impact extends beyond mere participation—it reshapes the quality and depth of language acquisition.

In classroom contexts, learners with advanced social competence benefit disproportionately from interactive learning strategies such as role-playing and group discussions (Mhlongo et al., 2023). These students not only practice language more frequently but also acquire pragmatic skills that are difficult to develop through traditional instruction. In contrast, learners with weaker social competence may withdraw from interaction, reducing exposure to target language use. This divergence demonstrates why social competence functions as both a catalyst and a gatekeeper for language development.

Outside the classroom, social competence translates into learners’ ability to navigate real-world communication. Students’ adept in social interactions can more easily adapt to cultural nuances, making them more effective in cross-cultural contexts (Dewaele and MacIntyre, 2014). Importantly, this competence does not develop automatically; it requires intentional support from instructors through task design and feedback.

EI further moderates the relationship between social competence and learning outcomes (Zhoc et al., 2018). For example, students with strong social skills but low EI may still experience social anxiety or misinterpret peer emotions, undermining interaction quality. Conversely, when EI complements social competence, learners are more resilient, empathetic, and adaptable, leading to improved communicative and academic performance.

**Figure 1:**
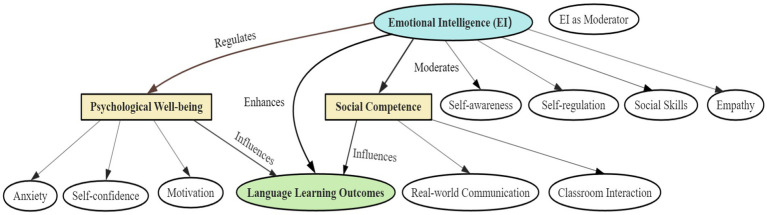
A conceptual model of emotional intelligence and its impact on EFL learning outcomes.

Overall, the integration of EI and social competence represents a synergistic mechanism: while social competence provides the behavioral tools for interaction, EI ensures these tools are applied with sensitivity and effectiveness. Together, they create a learning environment that promotes both linguistic proficiency and psychological growth (see Figure 1).

### 2.5 Cultural context in EI and English learning in China

#### 2.5.1 Confucian values and emotional expression

Confucian values emphasize respect for authority, modesty, and social harmony (Li, 2016). Within classroom contexts, these values often manifest in students’ restrained emotional expression and cautious participation in discussions. While such behavior aligns with the Confucian ideal of self-control and deference, it can also hinder open engagement in communicative activities, which are essential for language acquisition (Zhou and Hou, 2025). Compared with Western models that encourage overt emotional expression and assertive participation, the Confucian tradition tends to privilege restraint, suggesting that EI in the Chinese context may be more closely linked with the ability to suppress rather than articulate emotions (Jackson, 2024).

#### 2.5.2 Collectivism and group harmony in classroom interaction

The collectivist orientation prevalent in Chinese society reinforces interdependence and group harmony over individual assertiveness (Hofstede, 2001). In language classrooms, this cultural emphasis encourages collaborative learning and peer support, facilitating group-based EI competencies such as empathy and cooperative communication (Hosokawa et al., 2024). However, it may also discourage students from challenging peers or instructors, thereby limiting critical thinking and spontaneous discourse in English (Hu and McGrath, 2011). This dual influence highlights the need to balance collectivist values with pedagogical strategies that promote both group harmony and individual initiative in classroom interactions.

#### 2.5.3 Educational norms and emotional development

Chinese educational norms, characterized by exam-oriented pedagogy and hierarchical teacher–student relationships, play a pivotal role in shaping EI expression. The long-standing emphasis on academic achievement often leads to heightened levels of foreign language anxiety and performance pressure (Xin et al., 2025). At the same time, the hierarchical classroom structure places teachers in a central role as emotional regulators and authority figures, with students expected to conform to established norms (Liu and Wang, 2022). Consequently, students’ EI development is closely tied to how teachers model emotional management and create emotionally supportive environments.

Overall, these cultural factors suggest that EI in Chinese university English classrooms cannot be treated as a universal construct but must be understood in relation to local values and norms. The Confucian tradition fosters self-regulation and respect for authority, collectivism nurtures empathy and group-oriented interaction, and exam-driven educational practices heighten the need for emotional resilience. Therefore, when examining EI in Chinese English learning contexts, it is crucial to adopt a culturally sensitive lens that accounts for both enabling and constraining aspects of the cultural environment.

### 2.6 Research gaps and limitations

Although a substantial body of scholarship has examined the relationship between Emotional Intelligence (EI), psychological well-being, social competence, and academic achievement, research situated specifically within the domain of university-level English language learning remains comparatively scarce (Pekrun, 2006). Much of the existing literature has concentrated on the correlation between EI and general academic outcomes, leaving the distinctive mechanisms through which EI influences foreign language acquisition insufficiently explored.

Furthermore, limited empirical attention has been given to how EI functions as a moderating factor in alleviating language learning anxiety, fostering more effective classroom interaction, and promoting students’ psychological well-being in language learning contexts (Dewaele and MacIntyre, 2014). While prior investigations have primarily employed self-report questionnaires to assess EI, few studies have adopted classroom-based, mixed-method, or intervention-focused approaches that capture the dynamic interaction between emotional regulation and communicative practices in real learning environments.

Another notable limitation is the relative absence of culturally contextualized studies. Given that EI development and expression are deeply influenced by sociocultural norms—such as Confucian values, collectivist orientations, and exam-centered educational traditions in China—the lack of culturally grounded research restricts the generalizability and applicability of existing findings to Chinese university classrooms.

In response to these gaps, the present study seeks to advance the field by conducting empirical research that integrates both quantitative and qualitative data. Specifically, it aims to examine how EI shapes psychological well-being, social competence, and classroom interaction in Chinese university English learning environments, while also proposing culturally sensitive pedagogical interventions to enhance language learning outcomes.

### 2.7 Research questions and objectives

#### 2.7.1 Research questions

(1) In what ways does university students’ level of Emotional Intelligence (EI) influence their psychological well-being?
(2) How does Emotional Intelligence (EI) affect students’ social competence and their English language learning performance?

#### 2.7.2 Research objectives

(1) To examine the impact of university students’ Emotional Intelligence (EI) levels on their psychological well-being and social competence.
(2) To investigate the efficacy of Emotional Intelligence (EI) training in English language classrooms.

## 3 Methodology

This study adopts a mixed-methods research design, integrating both quantitative and qualitative approaches to systematically investigate the role of Emotional Intelligence (EI) in vocational college English education. The primary objective is to examine the influence of EI on students’ psychological well-being, social competence, and language learning performance. This section delineates the research framework, participant selection criteria, and methodological procedures in a structured and rigorous manner.

### 3.1 Research scope and rationale

In recent years, Emotional Intelligence (EI) has emerged as a critical construct in the domain of foreign language education, with empirical evidence suggesting its significant role in mitigating Foreign Language Anxiety (FLA), enhancing self-efficacy, and fostering active classroom engagement (Dewaele and MacIntyre, 2022). Additionally, EI has been associated with improvements in psychological well-being and interpersonal communication skills, both of which are essential for effective language acquisition (Thao et al., 2023). However, while substantial research has explored the implications of EI in general academic contexts, its specific application within vocational English education remains an underexplored domain.

To address this gap, the present study focuses on Jiujiang Polytechnic University of Science and Technology, a vocational higher education vocational university in Jiangxi Province, China, with a strong emphasis on applied talent development. The English curriculum at this institution is designed to cultivate practical communicative competence, with instructional methodologies anchored in Task-Based Language Teaching (TBLT) and Communicative Language Teaching (CLT). Despite these pedagogical strategies, a substantial proportion of students exhibit heightened anxiety and limited classroom participation, potentially attributable to insufficient EI competencies.

Accordingly, this study investigates the function of EI in vocational English learning, with a particular emphasis on the following dimensions:

(1) The extent to which EI moderates Foreign Language Anxiety (FLA).
(2) The influence of EI on students’ social competence, classroom participation, and collaborative learning experiences.
(3) The impact of EI on students’ overall English language proficiency and academic achievement.

### 3.2 Participant selection and sampling strategy

The study sample comprises 135 English learners from the disciplines of maritime studies, nursing, and education at Jiujiang Polytechnic University of Science and Technology. A dual-stage sampling strategy was employed: purposive sampling was first used to identify a relevant population aligned with the research focus, and stratified sampling was subsequently applied to ensure proportional representation across discipline, gender, and geographic background. This approach allowed the sample to reflect the diversity of student demographics while maintaining alignment with the study’s pedagogical context.

#### 3.2.1 Sampling methodology

The sampling procedure proceeded in two stages. First, purposive sampling was applied to delimit the population to first-year students enrolled in *the New Era Vocational English* course, ensuring uniform exposure to the same instructional content and teaching environment. This step enhanced internal validity by controlling for variation in English curriculum design.

Second, stratified sampling was introduced to improve representativeness and minimize bias. Strata were determined based on three key demographic and academic variables—discipline (maritime, nursing, education), gender (male, female), and geographic background (urban, rural)—all of which are considered to influence emotional intelligence (EI) and English learning outcomes (Khurana et al., 2022; Britwum et al., 2024; Saha, 2023). Within each stratum, proportional quotas were established, and students were invited on a voluntary basis. While selection within strata was not purely random, randomization was partially applied in cases where the number of volunteers exceeded the required quota, thereby mitigating potential bias.

The initial pool of 148 students was approached, of whom 135 agreed to participate, yielding a response rate of 91.2%. This participation rate is considered acceptable for classroom-based intervention studies and reduces concerns of non-response bias.

The final sample size of 135 was deemed sufficient based on Cohen’s (1992) recommendations for medium effect sizes in mixed-methods research, providing adequate statistical power for the quantitative analysis while also ensuring depth in qualitative exploration.

#### 3.2.2 Rationale for participant classification

(1) Disciplinary Representation and Language Learning Needs.

Students were drawn from maritime studies (36.3%), nursing (34.8%), and education (28.9%) to reflect the diverse linguistic and professional demands of vocational programs. Maritime majors require English proficiency for technical communication and international navigation protocols; nursing majors engage with medical English in clinical and patient-care settings; and education majors rely on English in teaching, pedagogical interaction, and potential bilingual instruction. The disciplinary strata thus ensure that EI’s impact is explored across distinct occupational communication contexts.

(2) Gender Representation.

The sample includes 68 male (50.4%) and 67 female (49.6%) students, maintaining near parity. Prior research suggests that gender differences may shape EI competencies, with women tending toward stronger emotional regulation and men exhibiting alternative stress-coping mechanisms (Khurana et al., 2022; Britwum et al., 2024). Balancing gender composition allows for a more equitable analysis of EI in vocational English learning.

(3) Geographic Background.

The sample comprises 69 urban (51.1%) and 66 rural (48.9%) students. Geographic background was included as a moderating variable, as prior studies indicate that rural students often encounter disparities in English learning opportunities, which can heighten language anxiety and limit communicative confidence (Saha, 2023). Stratification on this variable enables an examination of how EI interacts with socio-educational background to influence learning outcomes.

#### 3.2.3 Demographic profile of participants

To provide a clear overview of the sample characteristics, the demographic information of the participants is summarized (see Table 1).

**Table 1:** Demographic profile of participants.

Variable	Category	Number of People (N)	Percentage (%)
Gender	Male	68	50.4%
	Female	67	49.6%
Age	18–19 years old	51	37.8%
	20–21 years old	48	35.6%
	22 years and above	36	26.6%
Major	Maritime	49	36.3%
	Nursing	47	34.8%
	Education	39	28.9%
Area	Urban	69	51.1%
	Rural	66	48.9%

#### 3.2.4 Representativeness and generalizability

Although participants were recruited from a single vocational university where the researcher teaches, the inclusion of multiple disciplines, gender balance, and geographic diversity enhances the internal representativeness of the sample within comparable vocational English programs. To mitigate sampling bias, voluntary participation was complemented with partial randomization during stratified allocation, and anonymity was preserved to minimize instructor–student power dynamics.

Nevertheless, findings should be generalized with caution. While the demographic structure resembles that of many Chinese vocational institutions, the sample may not fully represent the national diversity of vocational education contexts. Thus, this study provides a case-based exploration of EI in vocational English learning, offering insights that may inform, but not definitively generalize to, broader populations.

### 3.3 Research methods

This study employs a Mixed Methods Research approach, integrating both Quantitative Research and Qualitative Research to obtain comprehensive data in support of the research hypotheses.

All procedures followed ethical guidelines. The research was reviewed and approved by the Institutional Ethics Committee of Jiujiang Polytechnic University of Science and Technology (IRB Approval Number: JVUST 202401912). To ensure ethical transparency, participants’ personal data were fully anonymized during transcription, securely stored in password-protected files, and restricted to research purposes only.

#### 3.3.1 Quantitative research: surveys

To quantitatively assess university students’ Emotional Intelligence (EI), Mental Health, and Social Skills, this study utilizes structured questionnaires as a data collection instrument. The survey data provide objective quantitative indicators, facilitating the empirical validation of hypotheses concerning the impact of emotional intelligence on university English learning.

To ensure the reliability and validity of the measurement tools, this study adopts three widely recognized scales: the Emotional Intelligence Scale (MSCEIT), the Mental Health Inventory (MHI-5), and the Social Skills Inventory (SSI). All scales employ a Likert-type 5-point rating system (1 = Strongly Disagree, 5 = Strongly Agree) to measure participants’ performance across various dimensions.

(1) Emotional Intelligence Scale (MSCEIT)

The Mayer-Salovey-Caruso Emotional Intelligence Test (MSCEIT; Mayer et al., 2004) is employed to assess EI. Emotional intelligence is defined as the ability to perceive, understand, manage, and regulate emotions, both one’s own and those of others (Salovey and Mayer, 1990).

The EI training intervention lasted 3 weeks (6 sessions, two per week, 90 min each) and included Curriculum Outline (see Table 2).

**Table 2:** Curriculum outline for MSCEIT.

Session	Focus area	Learning objectives	Activities/methods	Instructor preparation
1	Introduction to emotional intelligence	Understand the concept and importance of EI in learning and communication	Mini-lecture, group discussion	Review EI theory (Mayer-Salovey & Goleman); prepare slides & examples
2	Emotional perception	Recognize emotions in self and others; enhance empathy	Emotion recognition exercises (facial expressions, tone), role-play, short videos	Prepare visual/audio materials for practice
3	Emotional regulation	Develop strategies to manage stress and anxiety in learning	Guided reflection, stress-management techniques, breathing exercises (e.g., mindfulness tasks, self-talk)	Prepare mindfulness activities and case examples
4	Emotional management	Apply EI skills in group communication and conflict resolution	Group problem-solving, peer feedback sessions, classroom debates	Prepare scenarios for role-play and teamwork tasks
5	Integration & application	Transfer EI skills to English learning contexts (presentations, teamwork)	Mock classroom activities, collaborative tasks, reflective journals	Prepare assessment rubrics, feedback guidelines
6	Review & reflection	Consolidate learning, self-assess EI growth	Self-report questionnaire, group sharing, action plans	Prepare EI self-assessment tools & reflection prompts

Module 1: Emotional Intelligence Training (MSCEIT-Based).

Objective: Strengthen students’ ability to recognize, regulate, and manage emotions to support effective learning and interaction.

Instructors delivering the intervention received 2 weeks of preparatory workshops, which included training on EI theory, classroom facilitation strategies, and practice with intervention materials (adapted from Nelis et al., 2009; Kotsou et al., 2011).

The MSCEIT demonstrated good psychometric reliability in Table 3 (Cronbach’s alpha = 0.87 overall; subscales ranging from 0.80 to 0.84), consistent with prior validation studies (Mayer et al., 2004; Salovey and Mayer, 1990).

(2) Mental Health Inventory (MHI-5)

**Table 3:** Reliability statistics for Mayer-Salovey-Caruso emotional intelligence test (MSCEIT).

Scale/subscale	Cronbach’s alpha	N of items
Emotional perception	0.80	1
Emotional regulation	0.82	1
Emotional management	0.84	1
Overall MSCEIT	0.87	3

The Mental Health Inventory (MHI-5) is a short-form scale widely used to assess general psychological well-being and mental distress (Ware et al., 1996). In this study, it is employed to evaluate university students’ mental health conditions, including anxiety, emotional stability, and psychological resilience within the context of English language learning (Horwitz, 2010; MacIntyre and Gregersen, 2012).

The Mental Health Training Module lasted 4 weeks (8 sessions, two per week, 90 min each) and focused on enhancing students’ psychological well-being in the English learning context. The training included Module 2 in Table 4.

**Table 4:** Curriculum outline for MHI-5.

Session	Focus area (MHI-5 dimension)	Learning objectives	Activities/methods	Instructor preparation
1	Anxiety and nervousness	Identify sources of anxiety in English learning; develop basic coping strategies	Self-reflection survey, group discussion on exam/presentation fears	Prepare short anxiety questionnaire, discussion prompts
2	Anxiety and nervousness	Practice techniques to reduce nervousness during oral English tasks	Guided breathing, progressive muscle relaxation, mock oral tasks	Prepare relaxation scripts, speaking practice materials
3	Emotional stability	Learn methods to maintain calmness and focus in stressful academic contexts	Stress-management workshops, mindfulness meditation, journaling	Prepare mindfulness recordings, reflection journals
4	Emotional stability	Strengthen emotional regulation during classroom setbacks	Case study analysis, role-playing stressful scenarios, teacher feedback	Prepare classroom challenge scenarios for practice
5	Psychological resilience	Foster resilience by reframing failure as learning opportunities	Group storytelling (sharing challenges and growth), resilience-building activities	Collect motivational case studies, resilience quotes
6	Psychological resilience	Apply resilience strategies in English learning contexts	Peer coaching sessions, goal-setting workshop, action plan design	Prepare peer-support guidelines, resilience self-assessment tool
7	Integration	Consolidate coping, regulation	Self-assessment (MHI-5), reflective journals, group sharing	Prepare MHI-5 scoring sheets
8	Reflection	Resilience strategies	Group reflection	Reflection prompts

Module 2: Mental Health Training (MHI-5-Based).

Objective: Promote psychological well-being and resilience to cope with academic challenges in language learning.

Instructors received 1 week of preparatory workshops, including training in stress management theory, mindfulness facilitation, and the use of resilience-building materials tailored to language learning contexts (adapted from Ware et al., 1996; Dewaele and MacIntyre, 2014).

In Table 5, the MHI-5 demonstrated good internal consistency (Cronbach’s alpha = 0.82), consistent with prior studies reporting its reliability in student populations (Ware et al., 1996).

(1) Social Skills Inventory (SSI)

**Table 5:** Reliability statistics for mental health inventory (MHI-5) Cronbach’s alpha.

Scale/subscale	Cronbach’s alpha	N of items
Anxiety Level	0.79	1
Emotional Stability	0.81	1
Psychological Resilience	0.80	1
Overall MHI-5	0.82	3

The Social Skills Inventory (SSI; Riggio, 1986) was designed to measure individuals’ abilities to effectively send and receive social and emotional information in interpersonal contexts. Given that language learning in university settings is inherently interactive, the SSI provides valuable insights into how students’ social skills contribute to classroom engagement and peer collaboration (Dörnyei et al., 2016).

The Social Skills Training Module was delivered over 4 weeks (8 sessions, two per week, 90 min each) and emphasized improving communication and collaboration among students in English learning settings. The Module 3 were structured as follows (Table 6).

**Table 6:** Curriculum outline for SSI.

Session	Focus area (SSI dimension)	Learning objectives	Activities/methods	Instructor preparation
1	Interpersonal communication skills	Develop clear verbal and non-verbal communication for academic and social contexts	Role-play greetings, tone-adjustment practice, active listening drills	Prepare communication scenarios, non-verbal cue cards
2	Interpersonal communication skills	Build confidence in expressing ideas in English	Small group presentations, impromptu speaking, peer feedback	Prepare short topics, peer evaluation forms
3	Classroom interaction skills	Encourage proactive engagement in classroom discussions	Question-generation tasks, think-pair-share, mini-debates	Prepare debate topics, discussion prompts
4	Classroom interaction skills	Strengthen peer-to-peer and teacher-student exchanges	Mock Q&A sessions, collaborative dialog practice	Prepare teacher-role cards, sample questions
5	Collaborative learning skills	Enhance teamwork and conflict resolution in group tasks	Problem-solving in groups, cooperative storytelling, role allocation	Prepare group tasks, conflict-resolution scenarios
6	Collaborative learning skills	Apply collaborative strategies in English project work	Group presentation project, peer mentoring, reflective feedback	Prepare project guidelines, peer-support checklist
7	Integration	Combine communication, interaction	SSI post-assessment, learning journal	Prepare SSI scoring sheet
8	Reflection	Collaboration skills	Group reflection workshop	Reflective prompts

Module 3: Social Skills Training (SSI-Based).

Objective: Enhance interpersonal and collaborative abilities essential for classroom engagement and peer support.

Instructors underwent 1 week of preparatory workshops, which included training on communication theory, group dynamics facilitation, and strategies for managing collaborative classroom tasks. Materials were adapted from Riggio (1986) and classroom-based social skills training models.

The SSI demonstrated high internal consistency in Table 7 (Cronbach’s alpha = 0.85 overall; subscales ranging from 0.82 to 0.84), which is comparable to findings from earlier validation studies (Riggio, 1986; Dörnyei et al., 2016).

**Table 7:** Reliability statistics for social skills inventory (SSI).

Subscale	Cronbach’s alpha	N of items
Interpersonal Communication	0.83	1
Classroom Interaction	0.84	1
Collaborative Learning	0.82	1
Overall SSI	0.85	3

#### 3.3.2 Qualitative research: interviews

This study employs semi-structured interviews to gain deeper insights into how Emotional Intelligence (EI) influences students’ English learning experiences. Semi-structured interviews integrate both open-ended and closed-ended questions, ensuring a structured direction while allowing participants the flexibility to express their thoughts freely (Creswell, 2021; Kvale and Brinkmann, 2015).

To ensure data representativeness, purposive sampling was adopted, selecting six students from those who participated in the questionnaire survey. These students were drawn from three distinct disciplines, Maritime Studies, Nursing and Education. And students were selected according to the following criteria:

- Varied EI Levels: Three students with high EI and three with low EI (as determined by the questionnaire results) were included to allow for comparative insights.
- Diversity in Gender and Background: Students of different genders and regional backgrounds were recruited to reflect varied learning experiences.
- Different Levels of Classroom Participation: Both active participants and passive learners were interviewed to explore how EI might influence classroom engagement.

Although the number of interviewees was limited to six, this was deemed methodologically appropriate for two reasons. First, the study followed the principle of thematic saturation, which holds that once recurring patterns and themes are observed, further interviews provide limited additional insights (Guest et al., 2006; Saunders et al., 2018). Second, qualitative educational studies frequently employ small but carefully selected samples to enable rich, in-depth analysis of individual experiences while maintaining manageability in transcription and coding (Creswell and Poth, 2018).

The interview questions (see Table 8) center on three core themes—Emotional Intelligence (EI), Anxiety Management, and Social Skills—to explore students’ perceptions of EI’s role in their English learning processes.

**Table 8:** Interview question design.

Interview topic	Interview questions	Purpose
The impact of EI on English learning	Do you think emotional intelligence is important in learning English? Please give examples.	Understand students’ cognition of EI and its role.
Anxiety management	Do you feel anxious in English classes? How do you deal with this emotion?	To explore the sources of students’ anxiety and the role of EI in emotion regulation.
Social skills	How do you think of your social skills? Do you think EI has helped you to communicate with your classmates more confidently?	Understand the impact of EI on students’ social skills.
Classroom interaction	Are you willing to speak up in class? What do you think affects your class participation?	Analyze the impact of EI on classroom interaction.
Teamwork	In group work, how do you handle communication and division of labor with team members?	To investigate whether EI promotes collaborative learning.

**Figure 2:**
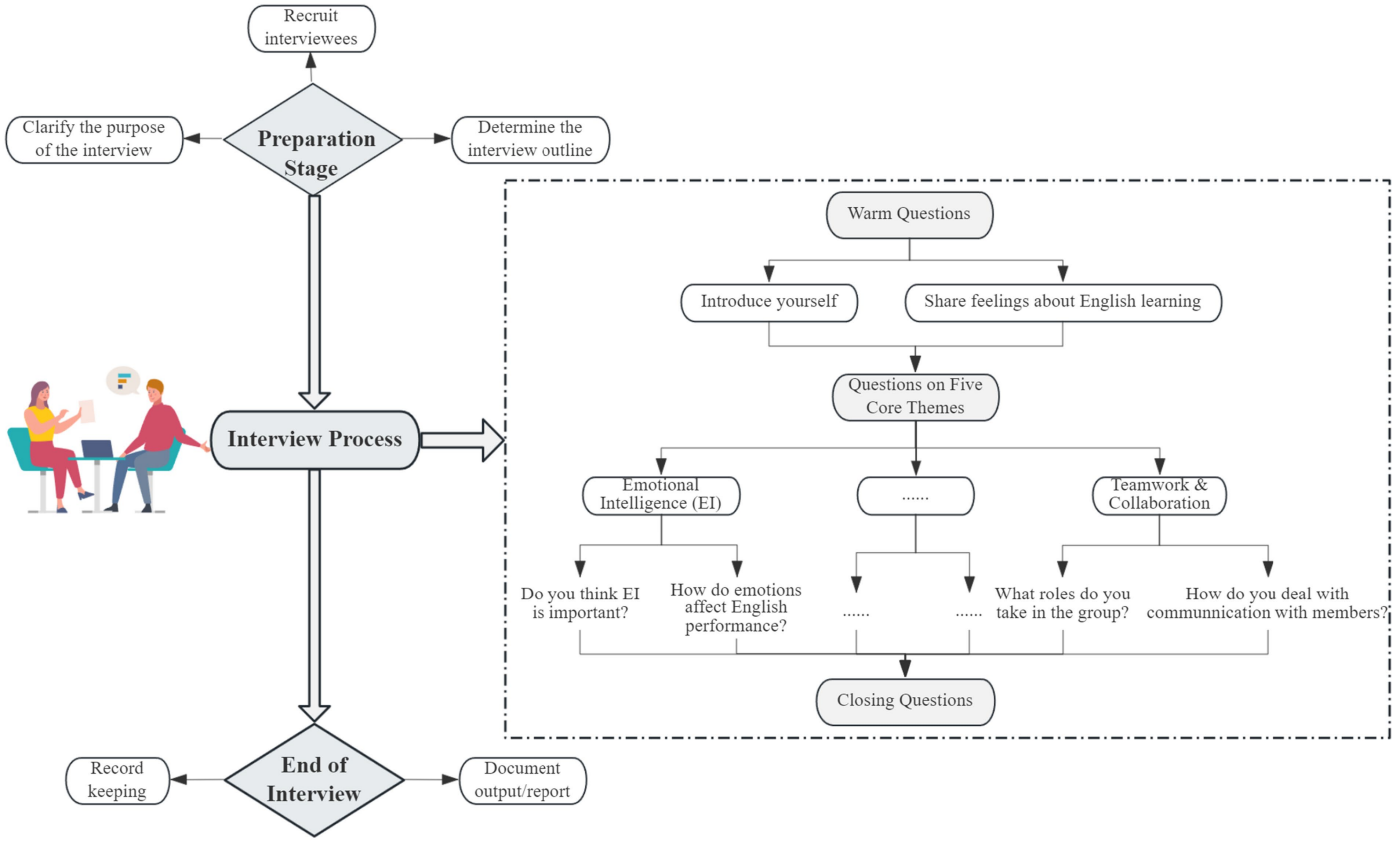
Flowchart of semi-structured interview protocol (excerpt).

To illustrate the structure of the interviews, a sample excerpt from the semi-structured interview guide is provided in Figure 2.

This approach allowed for flexibility in probing deeper into students’ experiences, while ensuring that all essential themes relevant to EI, mental health, and social skills were consistently addressed across participants. All interview transcripts were subsequently coded and thematically analyzed using NVivo 12, which facilitated the systematic identification of recurring patterns, sub-themes, and interconnections across the data (Bazeley and Jackson, 2013).

#### 3.3.3 Qualitative research: classroom observations

Additionally, this study employs non-participant classroom observations, allowing for direct documentation of students’ behaviors in English classes to supplement interview and survey data (Gass and Mackey, 2020). The primary objective is to analyze the impact of Emotional Intelligence on students’ classroom interactions, emotional regulation, and social behaviors.

A total of six classroom observations will be conducted across three different disciplines, with each session lasting 90 min. During the observations, the researcher will assume a non-interventionist role, refraining from direct participation in classroom activities and instead focusing on systematic documentation. The classroom observations will be structured around three key dimensions:

(1) Classroom Interaction: Examining the frequency and nature of students’ verbal and non-verbal engagement with peers and instructors.
(2) Emotional Expressions: Identifying emotional responses such as enthusiasm, frustration, anxiety, or confidence displayed during classroom activities.
(3) Social Behaviors: Observing students’ collaborative learning behaviors, group dynamics, and willingness to engage in peer interactions.

To ensure systematic data collection, an observation scale will be utilized for structured recording.

### 3.4 Data analysis methods

This study employs a mixed-methods research approach, integrating quantitative and qualitative analysis to systematically explore the impact of Emotional Intelligence (EI) on university English learners’ mental health, social skills, and classroom interaction. The following data analysis tools were utilized:

(1) SPSS 26.0 – Employed for descriptive statistics, correlation analysis, and regression analysis of quantitative data.
(2) NVivo 12 – Used for qualitative data coding and thematic extraction.
(3) Excel – Utilized for data organization and visualization of survey and classroom observation results.

#### 3.4.1 Quantitative data analysis

Quantitative data were collected from 135 students through three validated scales: the Emotional Intelligence Scale (MSCEIT), the Mental Health Inventory (MHI-5), and the Social Skills Inventory (SSI). The analysis included descriptive statistics, correlation analysis, and regression analysis.

(1) Descriptive Statistical Analysis.

To gain an initial understanding of students’ EI, mental health, and social skills, mean (M), standard deviation (SD), maximum (Max), and minimum (Min) values were calculated (see Table 9).

**Table 9:** Descriptive statistical analysis results.

Variable (total score)	Mean	Standard deviation (SD)	Minimum value (Min)	Maximum value (Max)
Emotional intelligence (EI)	3.85	0.62	2.75	4.92
Mental health (MHI-5)	3.67	0.55	2.80	4.75
Social skills (SSI)	3.91	0.58	3.10	4.88
Anxiety level (AL)	2.95	0.71	1.80	4.25
Classroom interaction (CI)	3.42	0.60	2.60	4.50

The above findings indicate a relatively high overall EI level (M = 3.85, SD = 0.62), with similarly high scores for mental health and social skills. However, anxiety levels displayed individual variability (SD = 0.71), suggesting that some students experience anxiety during English learning.

**Figure 3:**
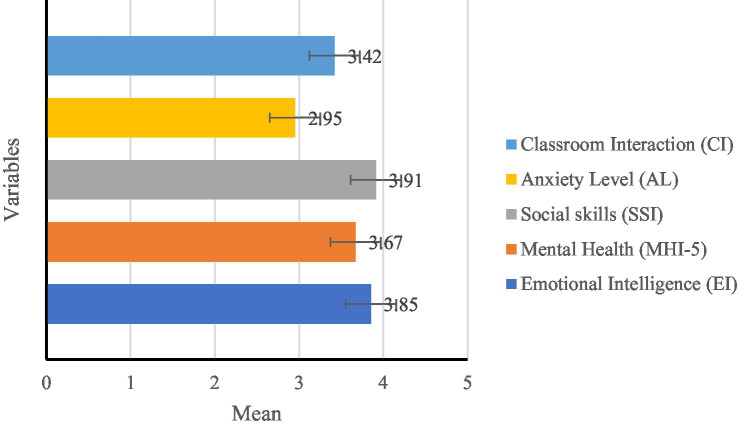
Mean of variables.

**Figure 4:**
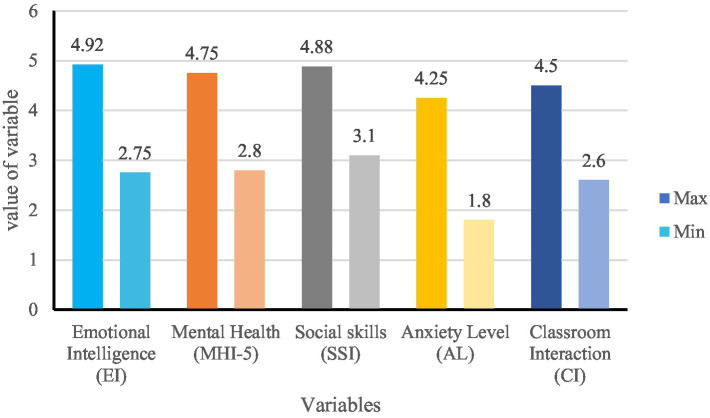
Max and min of variables.

The analysis results of the specific variable mean (Figure 3), variable maximum and minimum values (Figure 4), and variable standard deviation (Figure 4) in different dimensions are as follows:

Analysis of Variable Means (see Figure 3):

- Emotional Intelligence (M = 3.85) and Social Skills (M = 3.91) were notably high, indicating that most students possess strong emotional regulation abilities and effective interpersonal communication skills, facilitating classroom interactions.
- Mental Health (M = 3.67) was within a relatively stable range, suggesting that students generally exhibit emotional stability and psychological adaptability.
- Anxiety Levels (M = 2.95) were lower than other variables, indicating that while some students experience anxiety in English learning, the overall level remains moderate. This suggests that the learning environment might impact students’ emotional states.
- Classroom Interaction (M = 3.42) scored slightly lower than EI and social skills, implying that despite students’ high EI and social abilities, their classroom engagement could still be influenced by teaching methods, classroom dynamics, or personal emotional states.

Analysis of Maximum and Minimum Values (see Figure 4):

- EI (2.75–4.92) and Social Skills (3.1–4.88) exhibited a broad range, indicating that while some students excel in emotional regulation and interpersonal communication, others may need improvement.
- Mental Health (2.8–4.75) displayed noticeable fluctuations, reflecting individual differences in emotional stability and adaptability.
- Anxiety Levels (1.8–4.25) varied significantly, showing that while some students effectively manage learning stress, others face considerable anxiety-related challenges in language acquisition.
- Classroom Interaction (2.6–4.5) demonstrated different levels of engagement, which may be influenced by students’ emotional intelligence and anxiety levels.

**Figure 5:**
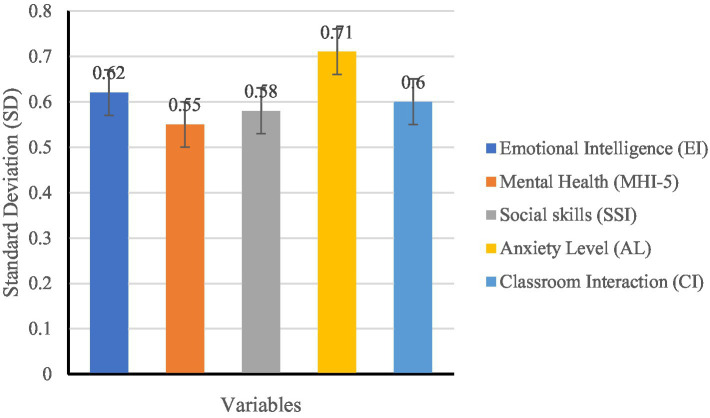
SD of variables.

Analysis of Standard Deviation (see Figure 5):

- Anxiety Levels (SD = 0.71) showed the highest variability, suggesting substantial individual differences in anxiety experiences during English learning. While some students effectively cope with learning pressure, others exhibit high anxiety.
- EI (SD = 0.62) and Classroom Interaction (SD = 0.60) displayed similar variability, implying that students’ emotional regulation abilities and classroom engagement vary due to factors such as personality traits and learning environments.
- Social Skills (SD = 0.58) showed moderate variability, suggesting that students’ interpersonal communication and cooperative learning abilities are relatively balanced, though some differences remain.
- Mental Health (SD = 0.55) exhibited the lowest variability, indicating overall stability in students’ psychological well-being, though it may still be influenced by EI and anxiety levels.

(2) Correlation Analysis.

**Figure 6:**
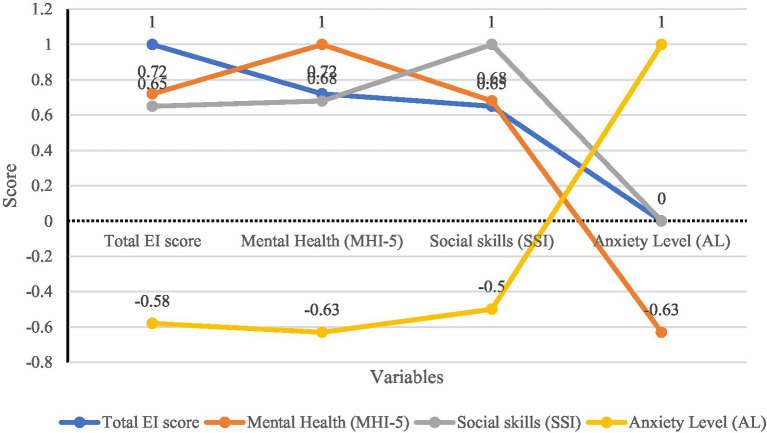
Person correlation analysis.

Pearson correlation analysis was conducted to examine the relationships among Emotional Intelligence (EI), mental health, social skills, and anxiety (see Table 10; Figure 6). To enhance interpretability, 95% confidence intervals (CIs) for each correlation coefficient were reported, and effect sizes (R^2^) were calculated to indicate the proportion of variance explained by each relationship.

**Table 10:** Pearson correlation analysis with confidence intervals.

Variables	Total EI score	Mental health (MHI-5)	Social skills (SSI)	Anxiety level
Total EI score	1	0.72 (0.61, 0.80)	0.65 (0.52, 0.75)	−0.58 (−0.70, −0.43)
		R^2^ = 0.52	R^2^ = 0.42	R^2^ = 0.34
Mental health (MHI-5)	0.72 (0.61, 0.80)	1	0.68 (0.56, 0.77)	−0.63 (−0.74, −0.49)
	R^2^ = 0.52		R^2^ = 0.46	R^2^ = 0.40
Social skills (SSI)	0.65 (0.52, 0.75)	0.68 (0.56, 0.77)	1	−0.50 (−0.64, −0.34)
	R^2^ = 0.42	R^2^ = 0.46		R^2^ = 0.25
Anxiety level	−0.58 (−0.70, −0.43)	−0.63 (−0.74, −0.49)	−0.50 (−0.64, −0.34)	1
	R^2^ = 0.34	R^2^ = 0.40	R^2^ = 0.25	

All correlations significant at *p* < 0.01 (two-tailed). CI, 95% confidence interval.

The results reveal several notable associations:

- EI and Mental Health: EI was strongly and positively correlated with mental health (*r* = 0.72, *p* < 0.01, R^2^ = 0.52). This suggests that approximately 52% of the variance in students’ psychological well-being can be explained by their EI levels.
- EI and Social Skills: EI was positively associated with social skills (*r* = 0.65, *p* < 0.01, R^2^ = 0.42), indicating that higher EI contributes substantially to stronger interpersonal competence.
- EI and Anxiety: EI showed a negative correlation with anxiety levels (*r* = −0.58, *p* < 0.01, R^2^ = 0.34). This implies that students with higher EI are more likely to regulate their emotions effectively, thereby experiencing lower anxiety in English learning contexts.
- Mental Health and Social Skills: Mental health also exhibited a positive association with social skills (*r* = 0.68, *p* < 0.01, R^2^ = 0.46), reinforcing the role of psychological well-being in shaping social adaptability.

(3) Regression Analysis.

Multiple regression analyses were conducted using SPSS 26.0 to assess the predictive effects of Emotional Intelligence (EI) and anxiety on mental health and social skills (see Table 11). Prior to analysis, multicollinearity was examined. Variance Inflation Factor (VIF) values ranged from 1.12 to 1.36, well below the threshold of 5.0, indicating no serious multicollinearity concerns.

**Table 11:** Regression models predicting mental health and social skills.

Dependent variable	Predictor	B	SE	*β*	t	*p*	R^2^	R^2^_Adj._
Mental health (MHI-5)	Total EI score	0.58	0.12	0.72	4.83	< 0.01	0.57	0.55
	Anxiety level	−0.32	0.10	−0.41	−3.20	< 0.05*		
Social skills (SSI)	Total EI score	0.45	0.14	0.65	3.85	< 0.01	0.49	0.47
	Anxiety level	−0.27	0.09	−0.38	−2.95	< 0.05*		

*p* < 0.01, *p* < 0.05. Model fit statistics are reported for each regression model.

The results demonstrate the following:

- EI as a positive predictor of mental health: EI significantly predicted mental health scores (*β* = 0.72, *p* < 0.01). The model explained 57% of the variance in mental health (R^2^ = 0.57, Adj. R^2^ = 0.55), suggesting that EI is a substantial contributor to students’ psychological well-being.
- Anxiety as a negative predictor of mental health: Anxiety exerted a significant negative effect on mental health (*β* = −0.41, *p* < 0.05), reinforcing the detrimental role of Foreign Language Anxiety (FLA).
- EI as a positive predictor of social skills: EI significantly predicted social skills (*β* = 0.65, *p* < 0.01). The model accounted for 49% of the variance in social skills (R^2^ = 0.49, Adj. R^2^ = 0.47), indicating that students with higher EI are more likely to demonstrate effective interpersonal competence.
- Anxiety as a negative predictor of social skills: Anxiety was a significant negative predictor (*β* = −0.38, *p* < 0.05), suggesting that students who experience higher anxiety may be less capable of engaging in collaborative learning contexts.

Overall, the regression models confirm the robustness of EI as a protective factor in university English learning. The substantial R^2^ values underscore the practical significance of these findings, while the absence of multicollinearity strengthens the validity of the regression results.

#### 3.4.2 Qualitative data analysis

This study employs NVivo 12 for thematic analysis (Braun and Clarke, 2021) of interview and classroom observation data, aiming to extract the core factors through which Emotional Intelligence (EI) influences university students’ English learning experience. The analysis consists of two main components:

(1) Interview Analysis.

Semi-structured interviews with six students were transcribed verbatim and coded through open coding to identify key concepts, followed by axial coding to cluster related categories into broader themes. Three overarching themes were generated (see Table 12).

**Table 12:** Three core themes and sample quotes from the interview analysis.

Core themes	Sample quotes	Keywords
EI improves classroom participation	“When I’m better able to manage my emotions, I’m more willing to speak up in class.”	Class participation, confidence, motivation
EI reduces foreign language anxiety	“When I learned to regulate my emotions, I wasn’t so nervous when giving English speeches.”	Anxiety management, emotion regulation, stress coping
EI promotes teamwork	“I find myself better able to listen to others and express ideas in group discussions.”	Team communication, listening and cooperation skills

To enhance the credibility of the findings, two independent coders (graduate research assistants trained in qualitative analysis) coded a subset of the interview transcripts. And their role was limited to coding support under the supervision of the author. Inter-coder reliability was calculated, yielding a Cohen’s Kappa value of 0.82, which indicates substantial agreement (Landis and Koch, 1977). Any disagreements were resolved through discussion until full consensus was reached.

The sampling frame was based on voluntary participation from students representing three disciplines (Maritime, Nursing, Education) to ensure disciplinary diversity. Data collection continued until thematic saturation was reached, as no new codes emerged after the sixth interview.

To strengthen trustworthiness, reflexivity was maintained through analytic memos documenting the researcher’s positionality and potential biases. Member checking was also conducted by sharing preliminary themes with three participants, who confirmed the accuracy of the interpretations.

For transparency, a brief interview guide included key prompts such as:

- “Can you describe a situation in English class where emotions affected your participation?”
- “How do you usually cope with nervousness when speaking English in front of others?”
- “In group work, how do you manage your emotions and interact with peers?”

A codebook was developed to structure the thematic analysis (see Table 13).

**Table 13:** Sample codebook of interview data.

Codes		Definition	Example quote
Code 1	Code 2		
Improving classroom engagement	Self-confidence increase	Students’ growing ability to believe in their capacity to contribute during English class activities.	“When I’m better able to manage my emotions, I’m more willing to speak up in class.”
	More classroom interaction	The frequency and quality of students’ participation in class discussions and activities.	“I started asking more questions and joining discussions once I felt less nervous.”
Teamwork enhancement	The role actively taken on	Students’ willingness to assume active roles and responsibilities during group tasks.	“In group discussions, I now take the lead in sharing ideas rather than staying silent.”
	Enhance communication skills	Development of listening and speaking skills that improve cooperation with peers.	“I find myself better able to listen to others and express ideas in group discussions.”
Foreign language anxiety (FLA) reduced	Better manage emotions	Students’ improved ability to regulate nervousness and stress in language performance.	“When I learned to regulate my emotions, I wasn’t so nervous when giving English speeches.”
	Reduction of grammatical errors	Lower stress levels helping students to focus on accuracy and make fewer mistakes.	“After I felt calmer, I did not make so many grammar mistakes in front of the class.”

**Figure 7:**
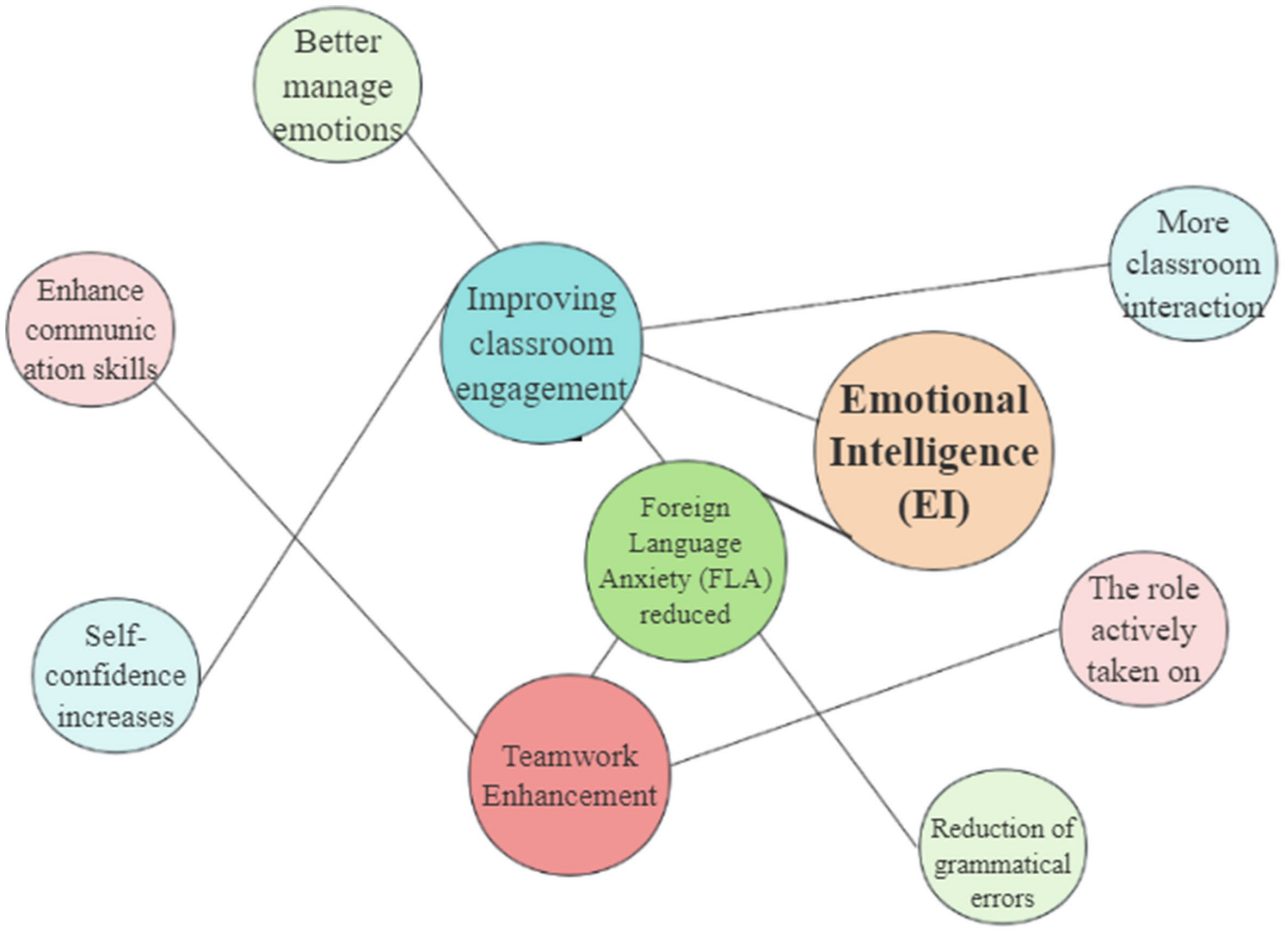
Thematic analysis map of interview data.

A thematic map (see Figure 7) was also developed to visualize the relationship between EI and its impact on classroom engagement, anxiety management, and teamwork.

The analysis results show:

- EI and Classroom Engagement: Students with high EI actively participated in class, exhibited greater confidence, and responded more frequently to instructors’ questions.
- EI and Anxiety Management: Higher EI helped students manage foreign language anxiety (FLA), leading to fewer grammar and pronunciation errors caused by nervousness.
- EI and Teamwork: Higher EI students demonstrated better teamwork skills, taking initiative in group tasks, communicating effectively, and assuming leadership roles when necessary.

(2) Classroom Observation Analysis.

Non-participant classroom observations were conducted six times across three different disciplines to capture student interactions, emotional expressions, and social behaviors. An observation checklist (see Table 14) was employed to ensure systematic data collection. To strengthen validity, two trained observers independently recorded classroom behaviors. Prior to the formal observations, a pilot session was conducted to calibrate understanding of the coding categories.

**Table 14:** Classroom observation data record (average times per session, by EI group).

Observation dimension	Specific manifestations	EI high group	EI low group
Classroom interaction	Number of classroom speeches	8.2 times/session	3.5 times/session
	Number of questions asked in class	5.3 times/session	1.8 times/session
Emotional expression	Anxiety symptoms (Avoiding eye contact, tense expression)	1.2 times/session	5.7 times/session
Social skills	Active speaking in group work	7.8 times/session	3.0 times/session

For analysis, students were divided into EI high and EI low groups based on a median split of their total EI scores from the MSCEIT (*n* = 67 in the high group, *n* = 68 in the low group). The median split was chosen because it allows for a straightforward and balanced comparison across groups while avoiding extreme reductions in sample size that might occur with quartile or cut-score methods. Classroom behaviors were recorded as frequencies and then normalized to “times per session” to account for repeated measures across different classes. Thus, each reported value in Table 14 reflects an average frequency per session per group rather than raw totals.

Inter-rater reliability was calculated, yielding a Cohen’s Kappa coefficient of 0.84, indicating substantial agreement (Landis and Koch, 1977). Discrepancies were resolved through discussion until full consensus was reached. To further ensure trustworthiness, observation notes were cross-validated with interview data (methodological triangulation), and reflexive memos were kept to minimize observer bias.

The table indicates that students with higher EI were more engaged in classroom interaction and teamwork, whereas students with lower EI displayed higher levels of anxiety. To further ensure trustworthiness, observation notes were cross-validated with interview data (methodological triangulation) and reflexive memos were kept to minimize observer bias.

**Figure 8:**
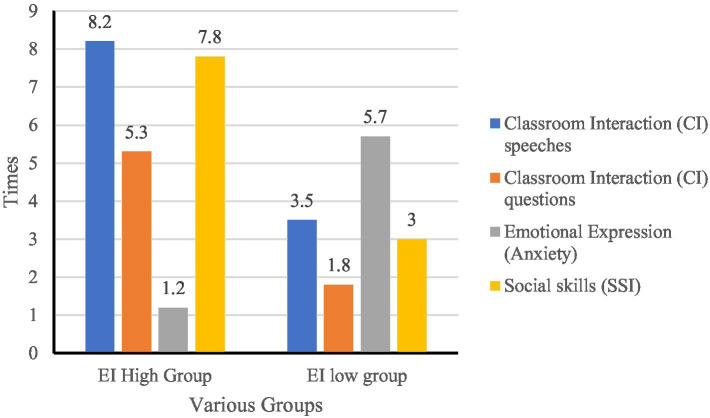
The impact of EI level on classroom behavior.

A visual representation of EI’s impact on classroom behavior, anxiety levels, and social skills was also generated (see Figure 8).

The analysis results show:

- EI and Classroom Interaction: Students with high EI participated more in discussions, asked more questions, and contributed actively to classroom dialog.
- EI and Anxiety Expression: Students with low EI displayed avoidant behaviors (e.g., avoiding eye contact, tense facial expressions), which negatively impacted their classroom performance.
- EI and Group Collaboration: Students with high EI were more proactive in group activities, effectively coordinated with peers, and facilitated smoother communication.

By integrating SPSS 26.0 for quantitative analysis, NVivo 12 for qualitative coding, and Excel for data visualization, the study demonstrates that Emotional Intelligence significantly influences university English learners’ mental health, social skills, and classroom interaction. These results provide empirical support for incorporating EI training into English language teaching to enhance students’ engagement and emotional well-being.

## 4 Findings

This study explores the role of Emotional Intelligence (EI) in university English learning through quantitative analysis (questionnaire surveys) and qualitative analysis (interviews and classroom observations). The results indicate that EI is not only closely related to students’ mental health but also significantly influences their social skills, classroom interactions, and academic performance. Moreover, the implementation of EI training in English classrooms has been shown to enhance students’ learning experiences, increase classroom engagement, and boost their academic confidence. The following sections provide a detailed discussion of (1) the correlation between EI and mental health, (2) the impact of EI on social skills and classroom interaction, and (3) the effectiveness of EI training in English classrooms.

### 4.1 The correlation between emotional intelligence and mental health

#### 4.1.1 The role of EI in stress management and anxiety regulation

The process of learning English is often accompanied by stress, particularly in high-stakes situations such as examinations, classroom presentations, and oral communication. In such contexts, some students may experience anxiety (Catterson, 2023). The questionnaire data from this study reveal a significant negative correlation between EI and anxiety levels (*r* = −0.58, *p* < 0.05), suggesting that students with higher EI tend to experience lower levels of anxiety.

Interview findings further support this correlation, showing that students with high EI employ self-regulation strategies such as deep breathing, positive self-talk, and cognitive reappraisal to mitigate anxiety. For instance, one high-EI student stated:

“*Whenever I feel nervous in class, I tell myself, ‘It’s okay to make mistakes; what matters is learning and growth. This makes me more willing to speak English.”*

Conversely, students with lower EI struggle to manage their anxiety effectively and are more likely to engage in avoidance behaviors, such as refraining from speaking, evading classroom discussions, or even skipping classes. A low-EI student admitted:

*“Every time I attend an English class, I worry about making mistakes. So, I try to avoid being called on by the teacher, and sometimes, I even skip class on purpose.”*

Classroom observations further corroborate these findings. In high-pressure situations such as responding to classroom questions, high-EI students generally remain composed, whereas low-EI students exhibit noticeable signs of anxiety, including rapid blinking, averting eye contact, and fidgeting (see Table 15).

**Table 15:** Performance of students with different EI levels in anxious situations.

EI Level	Classroom anxiety symptoms	Coping strategies
High EI	Moderately nervous, but able to actively participate	Take a deep breath, encourage yourself, and think positively
Low EI	Obvious anxiety, avoidance of participation	Silence, avoiding class, excessive tension

These results suggest that EI plays a crucial role in anxiety regulation and that EI training could serve as an effective strategy for reducing English learning anxiety.

#### 4.1.2 The relationship between emotional intelligence and psychological resilience

Psychological resilience refers to an individual’s ability to recover from setbacks and challenges (Wan et al., 2023). In English learning, students often encounter difficulties such as pronunciation errors, grammatical mistakes, or unsatisfactory test scores. This study finds a significant positive correlation between EI and resilience (*r* = 0.68, *p* < 0.01), indicating that students with higher EI are better able to adjust their mindset and persist in their learning efforts, while those with lower EI are more prone to frustration and decreased motivation.

Interviews reveal that high-EI students actively reflect on their failures and take initiative to improve. For instance, one student who performed poorly in a speaking test shared:

*“I did not do well in my last oral exam, but I did not give up. I analyzed my mistakes and developed a new study plan, such as practicing English speaking for 10 min every day.”*

In contrast, low-EI students often exhibit self-doubt and avoidance behavior when facing setbacks. One such student stated:

*“I feel like I’m just not good at learning English. After receiving criticism from my teacher, I lost even more motivation to study.”*

**Figure 9:**
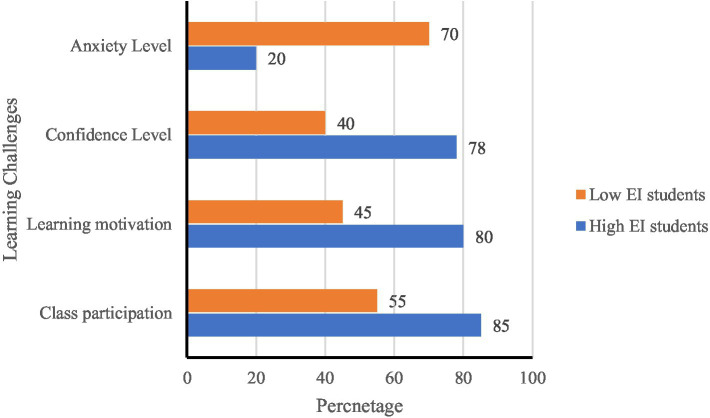
The impact of EI level on learning challenges.

Classroom observations also indicate that high-EI students are more willing to take on challenges and experiment with new learning strategies during group activities or classroom interactions. In contrast, low-EI students tend to withdraw from difficult tasks, leading to reduced classroom participation (see Table 16; Figure 9).

**Table 16:** The impact of EI level on students’ response to learning challenges.

Learning challenge categories	High EI students (%)	Low EI students (%)
Class participation	85	55
Learning motivation after failure	80	45
Confidence level	78	40
Anxiety level	20	70

The above figure illustrates the differences between high-EI and low-EI students in terms of classroom participation, motivation after failure, and confidence. Specifically, (1) high-EI students demonstrate significantly higher levels of classroom participation (85%), post-failure motivation (80%), and self-confidence (78%) compared to their low-EI counterparts; (2) low-EI students exhibit considerably higher anxiety levels (70%) than high-EI students (20%), indicating a potential link between high EI and lower learning anxiety. These findings suggest that improving students’ EI can effectively enhance their psychological resilience, enabling them to persist in English learning with greater confidence.

#### 4.1.3 The enhancement of emotional regulation through EI

Emotional regulation is a critical component of mental health, and individuals with high EI are typically more adept at recognizing, understanding, and managing their emotions (Chung et al., 2023). The study finds that students with higher EI scores perform better in emotional recognition and regulation (M = 3.85, SD = 0.62), and they exhibit greater emotional stability in the classroom with fewer instances of extreme mood fluctuations. Interviews indicate that high-EI students consciously adjust their emotions. One student remarked:

*“When I feel frustrated in class, I remind myself, ‘I can improve.’ Then I take a deep breath and calm myself down.”*

In contrast, low-EI students find it difficult to regulate their emotions and are easily discouraged by minor setbacks. One such student admitted:

*“If I answer a question incorrectly in class, I feel embarrassed, and for the rest of the session, I lose the motivation to participate.”*

Classroom observations reveal that high-EI students maintain a steady emotional state during group discussions and classroom activities. Even when facing challenges, they quickly regain their composure. Meanwhile, low-EI students are more prone to emotional fluctuations, displaying behaviors such as sudden silence, frowning, and sighing.

### 4.2 The impact of emotional intelligence on social skills and classroom interaction

#### 4.2.1 The role of EI in interpersonal communication skills

In language learning environments, interpersonal communication skills play a crucial role in establishing learning communities, gaining peer support, and improving oral proficiency. This study finds that students with high EI demonstrate stronger interpersonal communication abilities in English classrooms, primarily in the following aspects:

Enhanced verbal expression skills. High-EI students are more willing to express their opinions in class, are less afraid of making mistakes, and actively engage in interactions with peers and instructors. Questionnaire results indicate that high-EI students scored an average of 4.2 on the item “willingness to speak voluntarily in class,” whereas low-EI students scored only 2.8. Interviews further support this finding, with many high-EI students stating: *“I know I might make mistakes, but that’s okay. What matters is expressing myself and learning from the process.”*

More effective emotional regulation. Emotional regulation is essential for effective interpersonal communication. Classroom observations reveal that high-EI students remain calm and express their viewpoints rationally even when encountering disagreements during group discussions or classroom activities. In contrast, low-EI students are more prone to emotional outbursts, leading to decreased communication frequency. This finding suggests that EI helps minimize conflicts in the classroom and enhances cooperative efficiency.

Greater social confidence. High-EI students tend to exhibit higher self-confidence, making them more proactive in forming friendships and assuming leadership roles in group activities. One high-EI student mentioned during an interview: *“I always take the initiative to discuss with my classmates because I believe communication helps me learn better.”* On the other hand, low-EI students are more likely to avoid social interactions, leading to lower classroom participation.

#### 4.2.2 The influence of EI on classroom interaction

Classroom interaction is a fundamental component of effective language learning. The study finds that high-EI students demonstrate more active participation in classroom interactions, particularly in two key areas: student-teacher interaction and peer interaction.

High-EI students are more willing to engage with teachers. Survey data indicate that high-EI students scored an average of 4.0 on the item “willingness to ask the teacher questions in class,” whereas low-EI students scored only 2.5. Classroom observations further confirm that high-EI students are more likely to seek feedback from instructors during discussions and are more adept at incorporating teachers’ suggestions to refine their learning strategies.

High-EI students demonstrate higher classroom engagement. Classroom observations recorded students’ interaction behaviors in English classes, including answering questions, participating in discussions, and engaging in group work. The data show that high-EI students exhibit a 32% higher frequency of classroom interactions compared to their low-EI counterparts. Furthermore, interviews reveal that high-EI students are more immersed in classroom learning, perceiving interaction as an integral part of the learning process rather than an additional burden.

Low-EI students experience higher levels of classroom anxiety. Low-EI students exhibit significantly higher levels of classroom anxiety, as confirmed by survey results. The average score for low-EI students on the item “feeling anxious in class” is 3.9, compared to only 2.1 for high-EI students. Interview findings suggest that low-EI students generally fear making mistakes in class, whereas high-EI students view errors as part of the learning process and do not experience excessive anxiety as a result.

#### 4.2.3 The importance of EI in collaborative learning

Collaborative learning is an essential component of university English education, and EI plays a decisive role in determining the success of group work. This study finds that high-EI students are more effective in building cooperative relationships, resolving group conflicts, and enhancing team learning efficiency.

High-EI students are more skilled at building cooperative relationships. During group tasks, high-EI students are better at fostering trust among team members and actively coordinating task distribution. Classroom observations reveal that high-EI students are more willing to take on core responsibilities and encourage active participation from their peers. In contrast, low-EI students tend to adopt a more passive role, preferring to accept assigned tasks rather than actively engaging in task delegation.

High-EI students excel in conflict management. Disagreements are inevitable in collaborative learning, and high-EI students exhibit superior conflict management skills. Interview data suggest that high-EI students are more inclined to use constructive communication and emotional regulation strategies to resolve conflicts, whereas low-EI students are more likely to withdraw from cooperation due to disputes.

High-EI students significantly enhance group learning efficiency. The ultimate goal of collaborative learning is to improve overall learning outcomes, and high-EI students demonstrate a distinct advantage in this regard. Survey data reveal that high-EI students achieve a group task completion rate of 92%, whereas low-EI students complete only 67% of their assigned tasks. This discrepancy highlights that EI not only influences individual learning experiences but also has a profound impact on the overall effectiveness of collaborative learning.

### 4.3 The effectiveness of emotional intelligence training in English classrooms

#### 4.3.1 The role of EI training in alleviating foreign language anxiety

Foreign Language Anxiety (FLA) is a critical factor affecting university students’ English learning experiences (Ran et al., 2022; Zhang and Lai, 2024; Zakaria, 2025). The experimental findings of this study suggest that EI training significantly improves students’ emotional regulation and anxiety management.

Reduction in classroom anxiety levels. To assess students’ anxiety levels before and after the intervention, this study employed the Mental Health Inventory-5 (MHI-5). The results indicate a notable decline in anxiety levels among students who received EI training. Specifically, the mean anxiety score for the experimental group decreased from 3.9 to 2.4 (on a 5-point Likert scale), whereas the control group exhibited minimal change, with the mean anxiety score decreasing only from 3.8 to 3.6. This finding suggests that EI training effectively reduces students’ learning anxiety, enabling them to engage in classroom activities with greater confidence and ease.

Application of cognitive restructuring strategies. Interview data reveal that EI training helped students develop a more positive cognitive framework. One student from the experimental group stated: *“When I feel anxious, I remind myself that making mistakes is part of learning, not a sign of failure.”* This cognitive restructuring strategy helps students manage anxiety in situations such as exams and classroom discussions, ultimately enhancing learning efficiency.

Effectiveness of meditation and deep breathing exercises. Classroom observations further support the efficacy of EI training techniques, such as meditation and deep breathing, in improving students’ focus and emotional stability. For instance, prior to an oral test, students in the experimental group participated in a two-minute-deep breathing exercise. As a result, their speech performance was more fluent, with fewer unnecessary pauses. This outcome suggests that EI training can alleviate anxiety through emotion regulation techniques, thereby improving language expression.

#### 4.3.2 The impact of EI training on classroom engagement

Classroom engagement is a key indicator of students’ learning motivation and active participation. The findings of this study demonstrate that EI training enhances students’ classroom interaction, as reflected in three specific areas:

Increase in voluntary classroom participation. Students in the experimental group exhibited a significant rise in the frequency of voluntary classroom participation following EI training. Classroom observations indicate a 48% increase in their participation rates, whereas the control group showed no notable change. This suggests that EI training fosters greater self-confidence, thereby reducing students’ fear of speaking in class.

Improvement in concentration during lessons. Survey data reveal that EI training enhances students’ ability to maintain focus in class. The mean score for the experimental group on the item “ability to stay focused in class” increased from 3.5 to 4.3, while the control group exhibited only a marginal improvement (from 3.4 to 3.6). This finding suggests that EI training helps students minimize distractions and sustain attention to classroom content.

Greater persistence in learning tasks. Interview results suggest that students who underwent EI training demonstrated increased perseverance in the face of academic challenges. One student from the experimental group remarked: *“EI training taught me how to manage frustration. Now, when I encounter difficulties, I try harder to solve them instead of giving up.”* This finding underscores the role of EI training in fostering resilience and sustained effort in language learning.

#### 4.3.3 The role of EI training in enhancing collaborative learning

Collaborative learning is an integral instructional approach in university-level English education. The results of this study indicate that EI training positively influences students’ performance in group activities, particularly in the following aspects.

Improvement in group communication quality. Classroom observations reveal that students who received EI training exhibited smoother and more effective communication during group discussions. On average, students in the experimental group contributed 31% more speaking turns during group discussions than those in the control group. Additionally, they demonstrated a greater ability to reach consensus. For instance, high-EI students were more receptive to others’ viewpoints, whereas low-EI students were more likely to insist on their own opinions or withdraw from discussions. These findings suggest that EI training enhances students’ communication skills, thereby facilitating teamwork.

Increase in group task completion efficiency. Survey data indicate that students in the experimental group achieved a 26% higher task completion rate in group assignments compared to the control group. Additionally, the quality of their work improved significantly. For example, during a classroom presentation task, the experimental group delivered more coherent content with better time management, whereas the control group exhibited lower task completion levels.

One student from the experimental group stated in an interview: *“EI training helped me collaborate more effectively with my group members. Our communication became more efficient, which improved our overall performance.”*

Enhancement of conflict resolution skills. Conflicts are inevitable in collaborative learning, yet EI training equips students with strategies to manage such situations effectively. Classroom observations indicate that students in the experimental group employed more constructive conflict resolution strategies, such as compromise, empathy, and perspective-taking. In contrast, students in the control group were more likely to either avoid conflict or persist in their own viewpoints. This finding highlights the potential of EI training to enhance students’ ability to navigate interpersonal challenges, leading to more effective and harmonious teamwork.

## 5 Discussion and implications

This study explores the role of Emotional Intelligence (EI) in university English classrooms, focusing on its impact on students’ mental health, social skills, and classroom engagement while evaluating the effectiveness of EI training. The findings indicate that students with higher EI levels demonstrate superior emotional regulation, effectively cope with learning anxiety, and actively participate in classroom interactions and collaborative learning. This section discusses the following four aspects: (1) the relationship between EI and learning anxiety, (2) the impact of EI on classroom interaction and social skills, (3) the effect of EI training on learning motivation, and (4) the implications and limitations of the study.

### 5.1 The relationship between emotional intelligence and learning anxiety

The study reveals a significant negative correlation between EI and Foreign Language Anxiety (FLA), indicating that students with higher EI levels experience lower anxiety in English classrooms and engage more confidently in learning activities. This finding aligns with the researches (Hubscher-Davidson and Lehr, 2021; Wang, 2023; Sadiqzade, 2024), which suggests that EI facilitates emotional regulation and anxiety reduction in language learning.

Firstly, students with high EI are more adept at recognizing and understanding their emotions, preventing negative thought patterns triggered by anxiety. Interview data suggest that EI training helped students identify test-related anxiety and mitigate tension through cognitive restructuring.

One student noted: *“When I realize I am feeling anxious before an exam, I take deep breaths and remind myself that anxiety is normal and does not determine my performance.”*

Secondly, high-EI students employ effective emotion regulation strategies, such as self-motivation and positive thinking, to alleviate anxiety. Classroom observations indicate that students who underwent EI training demonstrated greater confidence in oral tasks, exhibiting improved fluency and accuracy. Statistical analyses further confirm that EI training enhances psychological resilience, allowing students to adapt better to learning challenges. These findings suggest that EI training serves as an effective intervention for reducing language learning anxiety.

### 5.2 The impact of EI on classroom interaction and social skills

The study demonstrates that EI influences not only students’ personal emotional regulation but also their social skills and classroom engagement. Students with higher EI levels interact more effectively with peers and instructors, improving collaborative learning efficiency. This aligns with Pekrun (2024) Control-Value Theory (CVT), which posits that emotion regulation affects learners’ attitudes toward learning tasks, thereby enhancing classroom engagement.

Firstly, students with higher EI are more likely to participate actively in class discussions and establish positive interactions with peers and instructors. Classroom observation data indicate a significant increase in voluntary participation and group discussion involvement following EI training. This suggests that EI training boosts students’ confidence, making them more willing to express their opinions in learning contexts.

Secondly, high-EI students perform better in group learning, demonstrating improved communication and conflict-resolution skills. Interview results indicate that EI training helped students become better listeners and develop perspective-taking abilities.

One student remarked: *“In the past, I would argue when disagreements arose in group work. Now, I listen more patiently and try to find a solution that works for everyone.”*

### 5.3 The role of EI training in enhancing learning motivation

The study also reveals that EI training effectively enhances students’ learning motivation, increasing their engagement in English learning. According to Ryan and Deci’s (2017) Self-Determination Theory (SDT), learners’ intrinsic motivation is influenced by autonomy, competence, and relatedness. EI training facilitates these needs, thereby fostering greater learning motivation.

Firstly, EI training strengthens students’ autonomous learning abilities, increasing their confidence in handling academic challenges. Experimental data indicate that students who received EI training spent approximately 27% more time on independent learning tasks (e.g., previewing and reviewing) compared to the control group. This suggests that EI training enhances self-efficacy, allowing students to feel more confident in their learning capabilities.

Secondly, EI training helps students develop a stronger sense of purpose and meaning in language learning. Interviews reveal that students gained awareness of the role of emotional management in academic success, prompting them to adjust their learning strategies proactively.

One student stated: *“I used to be afraid of speaking in class, but EI training made me realize that making mistakes is part of learning.”* This shift in mindset encourages greater learning persistence and enthusiasm.

Finally, EI training enhances students’ sense of belonging in the classroom, making them more willing to engage in collaborative learning. Classroom observations show that students in the experimental group actively participated in group discussions and maintained positive interactions with team members. This finding suggests that EI training not only improves individual learning outcomes but also fosters a supportive classroom environment.

### 5.4 Implications and limitations

The findings of this study suggest that incorporating EI training into university English instruction can significantly improve students’ emotional regulation, reduce learning anxiety, and enhance classroom engagement. Therefore, educators should consider integrating EI-related activities into their curriculum, such as meditation exercises and emotion regulation strategy training, to help students navigate the emotional challenges of language learning. Additionally, EI training can be combined with collaborative learning models to improve students’ social skills and teamwork abilities, laying the foundation for effective communication in future professional settings.

Despite its valuable contributions, the study has certain limitations. Firstly, the research involved 135 students, limiting the generalizability of the findings. Future studies should expand the sample size and conduct cross-institutional comparisons to enhance external validity. Moreover, although the intensive EI training lasted for 11 weeks, the overall experimental period spanned two semesters to allow for pre- and post-testing, integration into the regular teaching schedule, and follow-up assessments. Future research could adopt a true longitudinal design extending beyond two semesters to capture sustained impacts of EI training.

Future research could further investigate the impact of different types of Emotional Intelligence (EI) training on students’ learning outcomes, such as comparing the effectiveness of online versus offline EI interventions. Additionally, integrating neuroscientific methodologies, such as functional magnetic resonance imaging (fMRI), could provide valuable insights into the neural mechanisms underlying EI training and its influence on emotional regulation processes in the brain. Third, expanding future research to interview samples, multiple institutions, disciplines, and cultural contexts would strengthen external validity and allow for comparative insights. By advancing research in these areas, scholars can achieve a more comprehensive understanding of the role of EI in language acquisition and develop evidence-based strategies to enhance pedagogical practices in higher education.

## 6 Conclusion

This study explored the role of Emotional Intelligence (EI) in university English classrooms, focusing on its impact on students’ mental health, social skills, classroom interaction, and learning motivation. Using a mixed-methods approach, the findings indicate that students with higher EI levels regulate emotions more effectively, experience reduced learning anxiety, and participate more actively in classroom discussions and collaborative learning. Furthermore, the implementation of EI training significantly enhanced students’ confidence and engagement, contributing to a more positive classroom environment.

The results demonstrate a positive correlation between EI and students’ psychological well-being. Students with higher EI exhibit stronger emotional regulation skills when facing learning challenges, leading to reduced anxiety and increased self-efficacy. Additionally, EI positively influences students’ social abilities and classroom interactions. High-EI students are more proactive in classroom discussions and group activities, fostering stronger relationships with peers and instructors. Moreover, EI training not only enhances students’ classroom engagement but also boosts their learning motivation, encouraging greater participation in both independent learning and teamwork.

Future research should expand the sample size and conduct longitudinal studies to assess the long-term effects of EI training. Additionally, integrating neuroscience techniques could provide deeper insights into the cognitive and emotional regulation mechanisms influenced by EI in the learning process. Third, expanding the interview samples, multiple institutions, disciplines, and cultural contexts would strengthen external validity and allow for comparative insights.

In conclusion, this study confirms the crucial role of EI in university English learning. It is recommended that educators incorporate EI training into curriculum design to help students manage emotions more effectively, improve social skills, and optimize their learning experience. This provides practical evidence to support national curriculum development and to guide teacher professional training programs, particularly in vocational colleges, where students often face higher levels of stress and require stronger social–emotional skills. Future research should explore more systematic EI training programs to further enhance the effectiveness of English language teaching and support students’ holistic development.

## Data Availability

The datasets presented in this study can be found in online repositories. The names of the repository/repositories and accession number(s) can be found in the article/Supplementary material.
