# Supplementary material for: Emotional intelligence and holistic student development: an assessment of psychological and social efficacy in vocational university English education
Source: Front Psychol. 2025 Dec 18;16:1664645. doi: 10.3389/fpsyg.2025.1664645 (PMC12756152; doi:10.3389/fpsyg.2025.1664645)
Supplement: Supplementary file 1 [file Data_Sheet_1.PDF]

Appendix I: Student Questionnaire

Emotional Intelligence and Holistic Student Development: An Assessment of Psychological and Social Efficacy in Vocational University English Education

Dear Participant,

Greetings! This questionnaire is part of a research study exploring the relationship between Emotional Intelligence, Mental Health, Social Skills, and English language learning among university students. Your responses will remain anonymous and confidential. Please read each statement carefully and select the response that best describes your experience. Thank you very much for your cooperation and support!

Instructions: Based on your actual classroom experience, please choose the option that best reflects your situation. All items adopt a 5-point Likert scale:
(1 = Totally Disagree, 2 = Disagree, 3 = Neutral, 4 = Agree, 5 = Totally Agree)

Section I: Demographic Information

Gender:

Male□ Female□

Grade Level:

Freshman□ Sophomore□ Junior □ Senior□

Section II: Emotional Intelligence Scale (MSCEIT)

Test Dimensions	Topic	Details	Rating (1 = totally disagree, 5 = totally agree)
**Emotional Perception**	EI1	I can easily recognize changes in other people's emotions.	☐1☐2☐3☐4☐5
	EI2	I can accurately understand my current emotional state.	☐1☐2☐3☐4☐5
	EI3	I can tell how people feel by their facial expressions.	☐1☐2☐3☐4☐5
**Emotional Regulation**	EI4	I am able to control my emotions well when facing stress.	☐1☐2☐3☐4☐5
	EI5	I can quickly adjust my emotions to suit different learning and social situations.	☐1☐2☐3☐4☐5
	EI6	When faced with challenges, I am able to respond with a positive attitude.	☐1☐2☐3☐4☐5
**Emotional Management**	EI7	I can use emotional intelligence effectively to build good relationships with others.	☐1☐2☐3☐4☐5
	EI8	I am able to encourage positivity in others while working in a team.	☐1☐2☐3☐4☐5
	EI9	I can flexibly use my emotional intelligence in complex social situations.	☐1☐2☐3☐4☐5

Section III: Mental Health Inventory (MHI-5)

Test Dimensions	Topic	Details	Rating (1 = totally disagree, 5 = totally agree)
**Anxiety**	MHI1	I often feel nervous and uneasy in English class.	☐1☐2☐3☐4☐5
	MHI2	Before English exams, I would feel extremely anxious, which would even affect my performance.	☐1☐2☐3☐4☐5
	MHI3	When I speak in English in class, I feel my heart racing and my palms sweating.	☐1☐2☐3☐4☐5
	MHI4	I am afraid of making mistakes in English grammar or pronunciation in front of my classmates.	☐1☐2☐3☐4☐5
	MHI5	When studying English, I often feel overwhelmed and have difficulty concentrating.	☐1☐2☐3☐4☐5
**Emotional Stability**	MHI6	I am able to stay calm and find solutions when facing the pressure of English learning.	☐1☐2☐3☐4☐5
	MHI7	Even if I encounter setbacks in my English learning, I can maintain a positive emotional state.	☐1☐2☐3☐4☐5
	MHI8	I rarely feel long-term frustration or loss due to learning English.	☐1☐2☐3☐4☐5
	MHI9	When the English learning task is heavy, I can regulate my emotions well and avoid anxiety.	☐1☐2☐3☐4☐5
	MHI10	When I was learning English, I was able to stay focused on the task without being distracted by negative emotions.	☐1☐2☐3☐4☐5
**Psychological Resilience**	MHI11	When I encounter difficulties in learning English, I will actively seek solutions instead of giving up.	☐1☐2☐3☐4☐5
	MHI12	I can accept that my English level is not good enough and I am willing to work hard to improve myself.	☐1☐2☐3☐4☐5
	MHI13	When I encounter challenges in learning English, I will continue to persevere and will not be easily discouraged.	☐1☐2☐3☐4☐5
	MHI14	I believe that with continued hard work, I can improve my English skills and overcome my learning barriers.	☐1☐2☐3☐4☐5
	MHI15	When faced with failure or low scores in English learning, I am able to quickly adjust my mindset and keep working hard.	☐1☐2☐3☐4☐5

Section IV: Social Skills Inventory (SSI)

Test Dimensions	Topic	Details	Rating (1 = totally disagree, 5 = totally agree)
**Interpersonal Communication**	SSI1	I can express myself confidently in English, even in front of strangers.	☐1☐2☐3☐4☐5
	SSI2	I can maintain good eye contact and body language when talking to others.	☐1☐2☐3☐4☐5
	SSI3	When I communicate with others, I am able to understand them accurately and respond appropriately.	☐1☐2☐3☐4☐5
	SSI4	I am willing to actively communicate with classmates or teachers in and outside the classroom to improve my English expression skills.	☐1☐2☐3☐4☐5
**Classroom Interaction**	SSI5	I am willing to take the initiative to answer the teacher's questions in class, even if my answers may not be perfect.	☐1☐2☐3☐4☐5
	SSI6	I enjoy participating in class discussions and expressing my opinions actively.	☐1☐2☐3☐4☐5
	SSI7	I can actively contribute my ideas to group tasks and work with team members to complete the tasks	☐1☐2☐3☐4☐5
	SSI8	I think classroom interaction is very helpful for my English learning and I am willing to take the initiative to participate.	☐1☐2☐3☐4☐5
**Emotional Management**	SSI9	I am willing to take initiative in group work and help others solve problems.	☐1☐2☐3☐4☐5
	SSI10	In team activities, I can effectively coordinate and organize team members to complete learning tasks.	☐1☐2☐3☐4☐5
	SSI11	I am able to actively listen to others' opinions in group discussions and put forward my own views based on them.	☐1☐2☐3☐4☐5
	SSI12	I think teamwork can enhance my learning interest and efficiency.	☐1☐2☐3☐4☐5

Would you be willing to participate in a brief interview? (If yes, please leave your contact info; it will remain confidential):

Yes☐ Contact Info: ___________ No☐

Thank you again for your participation! Your responses are of great value to this research!
