# Supplementary material for: Emotional intelligence and holistic student development: an assessment of psychological and social efficacy in vocational university English education
Source: Front Psychol. 2025 Dec 18;16:1664645. doi: 10.3389/fpsyg.2025.1664645 (PMC12756152; doi:10.3389/fpsyg.2025.1664645)
Supplement: Supplementary file 2 [file Data_Sheet_2.PDF]

Appendix II: Semi-structured Interview Outline

Research Topic:

Emotional Intelligence and Holistic Student Development: An Assessment of Psychological and Social Efficacy in Vocational University English Education

Introduction to the Interview:

Thank you for agreeing to participate in this interview. The purpose of our conversation today is to better understand your English learning experiences and how your emotional intelligence may influence your classroom engagement, anxiety levels, and social interactions. Please feel free to express your honest thoughts, and there are no right or wrong answers. Your responses will be kept confidential and used for academic research only.

Interview Duration: Approximately 30–45 minutes

Interview Format: Individual, in-person or virtual (audio-recorded with consent)

Section 1: General Perception of Emotional Intelligence (EI)

1. How would you define emotional intelligence in your own words?

Follow-up: Do you think you have high or low emotional intelligence? Why?

2. Do you think emotional intelligence plays an important role in English learning? Why or why not?

Can you share any examples from your own experience?

Section 2: Emotional Regulation and Anxiety Management

3. How do you usually feel during English classes? For example, do you feel nervous, relaxed, excited, or anxious?

Follow-up: Can you describe a specific moment when you felt strong emotions in English class?

4. If you experience anxiety or stress in class, how do you cope with it?

Do you use any strategies to calm yourself down or regain focus?

5. Have your emotional responses ever affected your English learning performance (e.g., test scores, speaking performance, motivation)?

If yes, in what ways?

Section 3: Social Skills and Communication

6. How would you describe your social skills in English class (e.g., communicating with peers or teachers)?

Do you find it easy or difficult to express yourself in English?

7. Do you think emotional intelligence helps you interact more confidently with others in English?

Follow-up: Can you share a situation where EI helped (or failed to help) you in a social or classroom setting?

Section 4: Classroom Participation and Engagement

8. Are you comfortable speaking up in English class (e.g., answering questions, giving presentations)? Why or why not?

9. What factors influence your participation in class? (e.g., teacher's attitude, classroom atmosphere, your own confidence or emotions)

Follow-up: Do you think emotional intelligence helps you stay engaged?

Section 5: Teamwork and Collaboration

10. How do you feel about working in groups during English class? Do you enjoy it or find it challenging? Why?

11. When working in a group, how do you usually manage communication and divide tasks with your team members?

Follow-up: What role do you usually take in the group—leader, helper, follower, etc.?

12. Do you think emotional intelligence plays a role in successful group work? How?

Closing Questions

13. Looking back on your experience, how do you think improving your emotional intelligence might benefit your English learning?

14. Is there anything else you'd like to share about your emotions, social interactions, or personal experiences in English learning?

Thank you again for your time and thoughtful responses. Your input is extremely valuable to this study.
