# Supplementary material for: Emotional intelligence and holistic student development: an assessment of psychological and social efficacy in vocational university English education
Source: Front Psychol. 2025 Dec 18;16:1664645. doi: 10.3389/fpsyg.2025.1664645 (PMC12756152; doi:10.3389/fpsyg.2025.1664645)
Supplement: Supplementary file 3 [file Data_Sheet_3.PDF]

Appendix III: Classroom Observation Checklist

Study Title:

The Role of Emotional Intelligence in Enhancing Psychological Well-being, Social Competence, and Language Learning Outcomes in Chinese Vocational College English Classrooms

Observation Objective: To systematically capture observable student behaviors associated with emotional intelligence, social interaction, emotional regulation, and classroom engagement in university English courses.

Observation Type: Non-participant structured observation

Observation Duration: 90 minutes per session

Observation Frequency: 6 sessions across three disciplines (maritime, nursing, and education)

I. Language Learning Engagement Indicators

Behavioral Indicator	Yes	No	Notes / Examples
Student responds to teacher’s questions in English voluntarily	☐	☐	
Student completes language tasks with sustained focus	☐	☐	
Student asks questions or seeks clarification in English	☐	☐	
Student demonstrates effort in pronunciation, vocabulary use, or writing	☐	☐	

II. Emotional Intelligence Indicators

Behavioral Indicator	Yes	No	Notes / Examples
Student recognizes and names their emotional states (e.g., anxiety, excitement) during tasks	☐	☐	
Student demonstrates emotional self-regulation (e.g., calming down when anxious)	☐	☐	
Student provides emotional support or empathy to peers (e.g., encouragement, understanding)	☐	☐	
Student remains emotionally composed in face of failure or public performance	☐	☐	

III. Social Competence Indicators

Behavioral Indicator	Yes	No	Notes / Examples
Student engages in peer-to-peer interactions (e.g., initiating conversations, answering questions)	☐	☐	
Student participates actively in group tasks and discussions	☐	☐	
Student shows cooperative behavior (e.g., turn-taking, listening, contributing ideas)	☐	☐	
Student demonstrates ability to manage interpersonal conflict or disagreement appropriately	☐	☐	

Additional Notes / Researcher Observations:

____
____
____
____

Observer: ___Qilin Xuan___________

Class Observed: __________________

Date: ___________________________

Session Number: __________________
