# Supplementary material for: Emotional intelligence and holistic student development: an assessment of psychological and social efficacy in vocational university English education
Source: Front Psychol. 2025 Dec 18;16:1664645. doi: 10.3389/fpsyg.2025.1664645 (PMC12756152; doi:10.3389/fpsyg.2025.1664645)
Supplement: Supplementary file 4 [file Data_Sheet_4.PDF]

Informed Consent Form for Research Participants

Project Title:

Emotional Intelligence and Holistic Student Development: An Assessment of Psychological and Social Efficacy in Vocational University English Education

Principal Investigator:

Full Name: Qilin Xuan

Institution Name: Jiujiang Polytechnic University of Science and Technology

Email Address: 499483172@qq.com

Phone Number: +86 19987842396

九江科技职业大学
3601020412114

Institutional Affiliation:

Research Unit: Jiujiang Polytechnic University of Science and Technology

1. Invitation to Participate

You are being invited to take part in a research study conducted by **Qilin Xuan** from **Jiujiang Polytechnic University of Science and Technology**. Before deciding whether to participate, it is important that you understand the purpose of the research, what your participation will involve, and how your rights will be protected.

2. Purpose of the Study

The purpose of this study is to investigate how emotional intelligence (EI) affects university students' mental health, social competence, and performance in English language learning. The findings will contribute to improving teaching strategies in vocational English education.

3. Procedures

If you agree to participate, you will be asked to:

- Complete a questionnaire related to emotional intelligence, psychological well-being, and social interaction in English classes (approximately 20–30 minutes);
- (If selected) Participate in a 30–45 minutes semi-structured interview about your learning experiences;

- (If selected) Be observed in a classroom setting by the researcher during English class (non-intrusive observation only).

All procedures will take place during the academic semester and within normal class or scheduled research sessions.

4. Voluntary Participation

Your participation in this study is completely voluntary. You may choose not to participate or withdraw at any point without any penalty or loss of benefits to which you are otherwise entitled. Refusal to participate will not affect your academic standing or your relationship with the university.

5. Risks and Discomforts

There are no known physical risks associated with this study. Some questions may address personal feelings or attitudes, which could cause mild emotional discomfort. You may skip any questions you do not wish to answer.

6. Benefits

While there are no direct personal benefits to you, your participation may help educators and researchers better understand how emotional intelligence impacts English language learning and student well-being. The study may lead to improvements in future teaching methods and student support services.

7. Confidentiality

All data collected in this study will remain strictly confidential. Your name will not appear on any research records. Instead, an ID number will be used to code your responses. All digital data will be stored on a secure, password-protected server, and physical records will be locked in a secure location. Only the researcher and authorized supervisors will have access to the data.

Results of this study may be published in academic journals. However, your identity will never be revealed.

8. Use and Retention of Data

The data collected will be stored for three years after publication, solely for academic review purposes, after which all personal data will be securely destroyed.

9. Questions and Concerns

If you have any questions about this study or your rights as a participant, you are encouraged to contact the Principal Investigator:

Full Name: Qilin Xuan

Institution Name: Jiujiang Polytechnic University of Science and Technology

Email Address: 499483172@qq.com

Phone Number: +86 19987842396

If you have concerns about your treatment or rights as a research participant, you may also contact the Institutional Ethics Committee of Jiujiang Polytechnic University of Science and Technology at +86 18210370436

Participant Declaration

I have read and understood the information above. I have had the opportunity to ask questions and have received satisfactory answers. I voluntarily agree to participate in this study.

Participant's Name: ______________________________

Participant's Signature: ___________________________

Date: ____________________

Researcher's Name: ___Qilin Xuan___________________

Researcher's Signature: ___________________________

Date: ____________________
