# Supplementary material for: Emotional intelligence and holistic student development: an assessment of psychological and social efficacy in vocational university English education
Source: Front Psychol. 2025 Dec 18;16:1664645. doi: 10.3389/fpsyg.2025.1664645 (PMC12756152; doi:10.3389/fpsyg.2025.1664645)
Supplement: Supplementary file 5 [file Data_Sheet_5.PDF]

Ethical Review Report

Project Title:

Emotional Intelligence and Holistic Student Development: An Assessment of Psychological and Social Efficacy in Vocational University English Education

Principal Investigator:

Full Name: Qilin Xuan

Institutional Affiliation: Jiujiang Polytechnic University of Science and Technology

Research Duration: September, 2024 – June, 2025

Institutional Review Board (IRB):

Name of Institutional Ethics Committee: Jiujiang Polytechnic University of Science and Technology

1. Ethical Considerations and Approval

This research project was reviewed and approved by the Institutional Ethics Committee of Jiujiang Vocational University of Science and Technology prior to the commencement of data collection. The study complies fully with the ethical standards for research involving human participants as outlined in the Declaration of Helsinki (2013), and adheres to institutional guidelines for the protection of human subjects.

The study received official approval under **IRB Approval Number: JVUST 202401912**.

2. Informed Consent

All participants were fully informed about the nature, scope, and purpose of the research prior to their participation. A comprehensive informed consent form was distributed in both written and verbal formats. The form clearly stated that participation was voluntary, and that participants had the right to withdraw from the study at any time without penalty. Informed consent was obtained from all participants prior to data collection, and written documentation of consent was securely stored.

3. Confidentiality and Anonymity

To protect participant privacy, anonymity and confidentiality were strictly maintained throughout the research process. Personal identifiers were removed from

all datasets, and participants were assigned unique codes to ensure that their identities could not be traced. All digital data were stored on password-protected devices, and any physical materials were secured in locked cabinets accessible only to authorized researchers.

Only aggregated results are reported in any publications or presentations, and no individual data are disclosed.

4. Risk Assessment and Participant Welfare

The potential risks associated with this study were assessed to be minimal. The study involved questionnaires, interviews, and classroom observations that focused on emotional intelligence, classroom behavior, and language learning. Participants were not exposed to any physical, psychological, or social harm. Psychological topics such as stress or anxiety were addressed sensitively, and participants were reminded that they could skip any questions they felt uncomfortable answering.

In the event that a participant exhibited signs of psychological distress during data collection, referral information for university counseling services was made readily available.

5. Data Management and Retention

All data were collected, stored, and processed in compliance with relevant data protection legislation, including the [specify local regulation, e.g., Personal Information Protection Law of the People's Republic of China or GDPR if applicable]. Data will be retained for a period of three years after publication for verification purposes and then permanently deleted or destroyed.

6. Research Integrity and Transparency

The researchers affirm that all procedures comply with established standards of research integrity, including transparency, honesty, and accountability in data collection, analysis, and reporting. No form of plagiarism, data fabrication, or manipulation was involved in any stage of this research.

This study has not been submitted to or published in any other journal, and all co-authors have reviewed and approved the final manuscript.

7. Declaration of Conflicts of Interest

The researchers declare no potential conflicts of interest in the conduct and dissemination of this study.

Submitted by:

Researcher: Qilin Xuan

Email Address: 499483172@qq.com

Date: October 2, 2025
